# ﻿Expanding the taxonomy of crab spiders (Araneae, Thomisidae) in Sumatra: a new genus, five new species, and regional records

**DOI:** 10.3897/zookeys.1241.148348

**Published:** 2025-06-13

**Authors:** Naufal Urfi Dhiya'ulhaq, Suresh P. Benjamin, Damayanti Buchori, Purnama Hidayat, Stefan Scheu, Jochen Drescher

**Affiliations:** 1 Animal Ecology, J.F. Blumenbach Institute of Zoology and Anthropology, University of Göttingen, Göttingen, Germany; 2 Species Obscura, Depok, Indonesia; 3 National Institute of Fundamental Studies, Kandy, Sri Lanka; 4 Department of Plant Protection, Faculty of Agriculture, IPB University, Bogor, Indonesia; 5 Centre for Transdisciplinary and Sustainability Sciences, IPB University, Bogor, Indonesia; 6 Centre of Biodiversity and Sustainable Land Use, University of Göttingen, Göttingen, Germany; 7 Senckenberg Museum for Natural Sciences Görlitz, Görlitz, Germany

**Keywords:** Arachnida, biodiversity, RTA-clade, Southeast Asia, taxonomy

## Abstract

The taxonomy of crab spiders (Thomisidae) has been the focus of many reviews, adding new genera such as *Ibana* and *Crockeria* while synonymising *Ascurisoma* with *Cebrenninus*, and describing many new species. A collection of crab spiders from Jambi Province (Sumatra, Indonesia) revealed further diversity, resulting in the description of a new genus, *Rangkayo* Dhiya’ulhaq & Benjamin, **gen. nov.**, and five new species: *Crockerianeofelis* Dhiya’ulhaq & Benjamin, **sp. nov.** (♂♀), *Ibanasvarnadvipa* Dhiya’ulhaq & Benjamin, **sp. nov.** (♂♀), *Phartaroseomaculata* Dhiya’ulhaq & Benjamin, **sp. nov.** (♀), *Rangkayohitam* Dhiya’ulhaq & Benjamin, **sp. nov.** (♂♀), and *Rangkayoperkaso* Dhiya’ulhaq & Benjamin, **sp. nov.** (♂♀). Additionally, new records of *Angaeuschristae* Benjamin, 2013 (♂), *Angaeusverrucosus* Benjamin, 2017 (♂), *Crockeriakinabalu* Benjamin, 2016 (♂), *Epidiuselongatus* Benjamin, 2017 (♂), and *Phartabimaculata* Thorell, 1891 (♂♀) are provided for Sumatra, as well as high-resolution images of *Cebrenninusmagnus* Benjamin, 2016 (♂) and *Cebrenninusrugosus* Simon, 1887 (♀). The present study highlights the considerable biodiversity of tropical crab spiders and underscores the importance of continued taxonomic and ecological research in Southeast Asia in general, and the Indonesian archipelago in particular.

## ﻿Introduction

The Thomisidae (Crab Spiders) in this study correspond approximately to the *Epidius* clades defined by Benjamin (2011) and Benjamin et al. (2008). Based on the data available at the time, these clades included the genera *Borboropactus* Simon, 1884, *Epidius* Thorell, 1877, *Pharta* Thorell, 1891, *Geraesta* Simon, 1889, and *Cebrenninus* Simon, 1887. *Ascurisoma* Strand, 1928 was also grouped within the *Epidius* clades. Their monophyly was supported by two proposed characteristics: the presence of a macro-trichobothrium on the palpal tibia (assumed to be lost in *Borboropactus*) and claw tufts extending from the tip of the claw towards the tarsus/metatarsus joint. Notably, *Borboropactus* was placed in its distinct clade by Benjamin et al. (2008), as it is unambiguously identifiable by a specialised sensory region on the dorsal surface of the tarsi (Benjamin 2011: fig. 24C, D).

Although *Borboropactus* is currently classified within the family Thomisidae, it shares several characteristics with members of the RTA clade (retrolateral tibial apophysis) not possessed by derived members of Thomisidae, such as the presence of cheliceral teeth, a median apophysis, a conductor, and epigynal teeth (Benjamin 2011; Ramírez 2014; Wheeler et al. 2017). A recent study suggests that *Borboropactus* is more closely related to Psechridae than to Thomisidae (Kulkarni et al. 2023).

*Ascurisoma* was later synonymised with *Cebrenninus* (Benjamin, 2016). *Angaeus* Thorell, 1881, and its junior synonym *Paraborboropactus* Tang & Li, 2009, were not included in any of the aforementioned phylogenetic studies. Subsequently, two new genera, *Ibana* Benjamin, 2014 and *Crockeria* Benjamin, 2016, were described. Collectively, these thomisid genera are now well-defined, with their composition and distribution relatively well-documented. However, as evidenced by this new collection from Jambi Province (Sumatra, Indonesia), the study of biodiversity continues to reveal unexpected findings.

In the present taxonomic contribution, we examine a collection of 32 specimens of thomisids of the *Epidius* clade belonging to the known genera *Angaeus*, *Cebrenninus*, *Crockeria*, *Ibana*, *Pharta*, and propose the new genus *Rangkayo* Dhiya’ulhaq & Benjamin, gen. nov. The specimens were collected by canopy fogging from 32 permanent research plots of the EFForTS project in Jambi, Sumatra (Drescher et al. 2016). In addition to the new genus *Rangkayo*, based on two species, we then describe three new species belonging to *Crockeria*, *Ibana*, and *Pharta*, highlighting the remarkable diversity of tropical crab spiders and underscoring the richness of Southeast Asia’s biodiversity.

## ﻿Materials and methods

### ﻿Sample collection

The specimens used here were part of a collection of more than half a million canopy arthropods (Pollierer et al. 2023), sampled in 2013 across 32 permanent research plots of the EFForTS (Ecological and Socioeconomic Functions of Tropical Lowland Rainforest Transformation Systems) project in the lowlands of Jambi Province, Sumatra, Indonesia (Drescher et al. 2016). EFForTS research plots were evenly distributed among four land-use systems (eight plots each): Lowland rainforest, ‘jungle rubber’ (extensively managed rubber agroforestry), and smallholder monocultures of rubber (*Heveabrasiliensis*) and oil palm (*Elaeisguineensis*). Canopy fogging was conducted by applying 50 ml of DECIS 25 (Bayer Crop Science; active ingredient deltamethrin, 25 g/L) dissolved in four litres of petroleum oil (‘white oil’) to three target canopies in each plot. Underneath each of the three target canopies, 16 square collection traps were placed, each measuring 1 m × 1 m, to which PE bottles with 100 ml 99.8% EtOH p.A. were attached. Two hours after applying the entire mixture of white oil and insecticide to the target canopies, the contents of the collection traps were combined into a single 1 L PE bottle per target canopy with fresh ethanol. Canopy arthropods were stored at -20 °C whenever possible, and subsequently sorted to orders and deeper taxa, including spiders. Overall, more than 10,000 spider individuals from more than 30 families and at least 400 species were found in the traps of two subplots per plot (Ramos et al. 2022). More details can be found in Drescher et al. (2016) concerning plot design and the EFForTS research framework, Pollierer et al. (2023) regarding the canopy fogging method, and Ramos et al. (2022) with regard to biodiversity patterns of the collected canopy spider community.

### ﻿Identification and photography

Specimens were examined under a ZEISS Stemi 2000 microscope. Female genitalia were excised from the specimen’s body and then cleared with 10% KOH for at least one hour to examine the internal copulatory organs. The specimens were measured and imaged using a KEYENCE VHX–7000 digital microscope system. The description of colouration is based on specimens preserved in ethanol. Measurements of legs are given as total length (femur, patella, tibia, metatarsus, tarsus). Missing legs or leg segments are marked with ‘-’, and legs with missing segments do not have their total length recorded. All measurements are in millimetres.

### ﻿Abbreviations

**AER** anterior eye row;

**ALE** anterior lateral eye;

**AME** anterior median eye;

**CO** copulatory openings;

**CD** copulatory ducts;

**FD** fertilisation ducts;

**MA** median apophysis of male palp;

**PER** posterior eye row;

**PLE** posterior lateral eye;

**PME** posterior median eye;

**RTA** retrolateral tibial apophysis of male palp;

**TA** tegular apophysis of male palp;

**VTA** ventral tibial apophysis of the male palp.

### ﻿Repositories

**GOET** Animal Ecology Group, Johann-Friedrich Blumenbach Institute of Zoology and Anthropology, Georg-August University of Göttingen, Göttingen, Germany

**MZB** Museum Zoologicum Bogoriense, Cibinong, Bogor, Indonesia

**SMF** Naturmuseum Senckenberg, Frankfurt am Main, Germany

**ZMH** Museum der Natur Hamburg, Hamburg, Germany

## ﻿Results

**Family Thomisidae Sundevall, 1833**

***Angaeus* Thorell, 1881**

### ﻿ Angaeus christae

Taxon classificationAnimaliaAraneaeThomisidae

Benjamin, 2013

32760BD6-D099-58AD-89E7-03445F980DAB

Figs 1 , 2

Angaeus christae Benjamin, 2013: 74, figs 3A, B, E, 5A–D.

#### Material examined.

Indonesia – Sumatra, Jambi Province • 1♂; Sarolangun, Air Hitam, Desa Baru; 02°04'36.0"S, 102°46'22.4"E; elev. 54 m; 27 Jun. 2013; J. Drescher leg.; canopy fogging in rubber plantation; GOET 2013_BR4.1_AraThom025N_001 (to be transferred to MZB).

#### Diagnosis.

**Figure 1.**
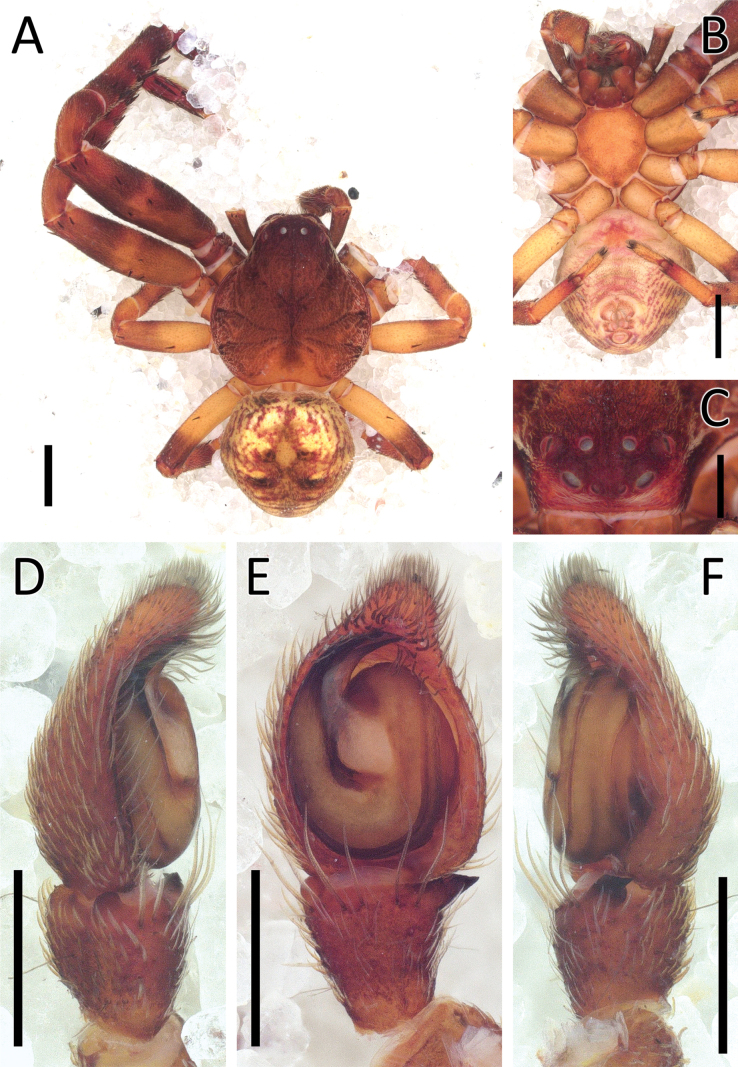
*Angaeuschristae* Benjamin, 2013, male (2013_BR4.1_AraThom025N_001) **A, B** habitus **A** dorsal view **B** ventral view **C** eye region, frontal view **D–F** left Palp **D** prolateral view **E** ventral view **F** retrolateral view. Scale bars: 1 mm (**A, B**); 0.5 mm (**C**); 0.2 mm (**D–F**).

**Figure 2.**
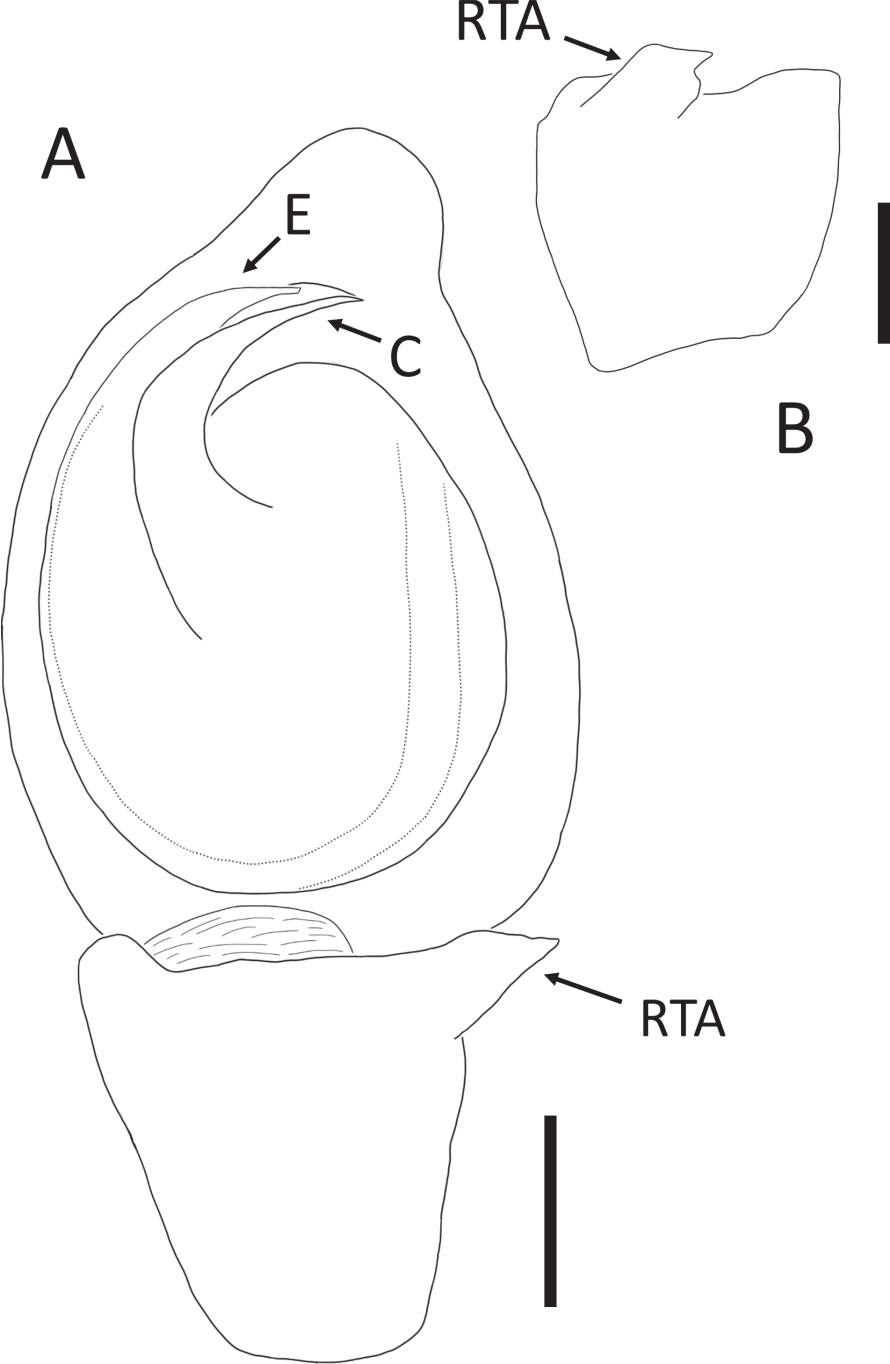
*Angaeuschristae* Benjamin, 2013, male (2013_BR4.1_AraThom025N_001) **A, B** left Palp **A** ventral view **B** tibia, retrolateral view. Abbreviations: C = conductor; E = embolus; RTA = retrolateral tibial apophysis. Scale bars: 0.2 mm.

Distinguished from all other congeners by the lack of MA in males (Figs 1D–F, 2), and the kidney-shaped spermatheca in females (Benjamin 2013: fig. 5C, D).

#### Description.

**Male** (2013_BR4.1_AraThom025N_001; Figs 1, 2). Total length 6.70. Carapace length 2.89; width 2.70. Abdomen length 3.81; width 2.25. Diameter of eyes: AME 0.08; ALE 0.17; PLE 0.13; PME 0.10. Interdistances between eyes: AME–AME 0.15; AME–ALE 0.08; ALE–ALE 0.44; PME–PME 0.24; PME–PLE 0.23; ALE–PLE 0.19; AME–PME 0.27; PLE–PLE 0.89. Leg measurements: leg I 9.10 (2.94, 1.19, 2.52, 1.62, 0.83); leg II 8.60 (2.84, 1.02, 2.35, 1.55, 0.84); leg III 4.26 (1.43, 0.56, 1.08, 0.67, 0.52); leg IV 4.62 (1.64, 0.54, 1.17, 0.77, 0.50). Carapace pear-shaped, dark reddish brown; AER recurved; PER recurved. Abdomen oval, yellow with streaks of red; cardiac mark drab yellow. Front legs much thicker than back legs, dark reddish brown with dark blotches; back legs lighter. Palp (Figs 1D–F, 2): cymbium 1.5 × length of tibia. Conductor curved, forming a sheath for the embolus. Embolus claw-shaped with a thick base. RTA short and stout, roughly triangular.

#### Distribution.

Indonesia (Sumatra: Jambi Province, new record); Malaysia (Borneo) Fig. 29.

#### Remarks.

Given the lack of MA and the oval abdomen shape of the males (rhomboid in females and all other congeners), it is possible that this species should belong in a different genus and that the female paratype described in Benjamin (2013) is in fact not conspecific with the holotype male. However, due to the lack of additional specimens, we refrain from making any taxonomic changes.

### ﻿ Angaeus verrucosus

Taxon classificationAnimaliaAraneaeThomisidae

Benjamin, 2017

E50C691C-4DC3-5C8D-8927-6CC77D4183E8

Figs 3 , 4

Angaeus verrucosus Benjamin, 2017a: 297, figs 1–14.

#### Material examined.

Indonesia – Sumatra, Jambi Province • 1♂; Batang Hari, Hutan Harapan Conservation Area; 02°10'42.4"S, 103°19'58.2"E; elev. 54 m; 3 Aug. 2013; J. Drescher leg.; canopy fogging in rainforest; GOET 2013_HF3.2_AraThom093N_001 (to be transferred to MZB).

#### Diagnosis.

See Benjamin (2017a).

#### Description.

**Figure 3.**
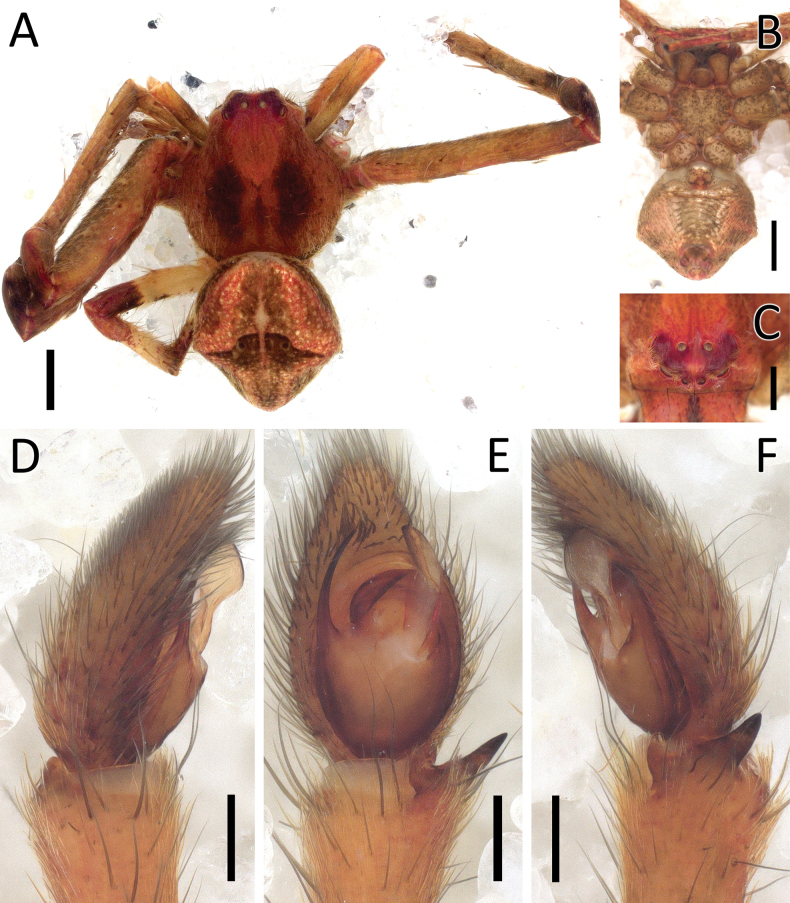
*Angaeusverrucosus* Benjamin, 2017, male (2013_HF3.2_AraThom093N_001) **A, B** habitus **A** dorsal view **B** ventral view **C** eye region, frontal view **D–F** left Palp **D** prolateral view **E** ventral view **F** retrolateral view. Scale bars: 1 mm (**A, B**); 0.5 mm (**C**); 0.2 mm (**D–F**).

**Figure 4.**
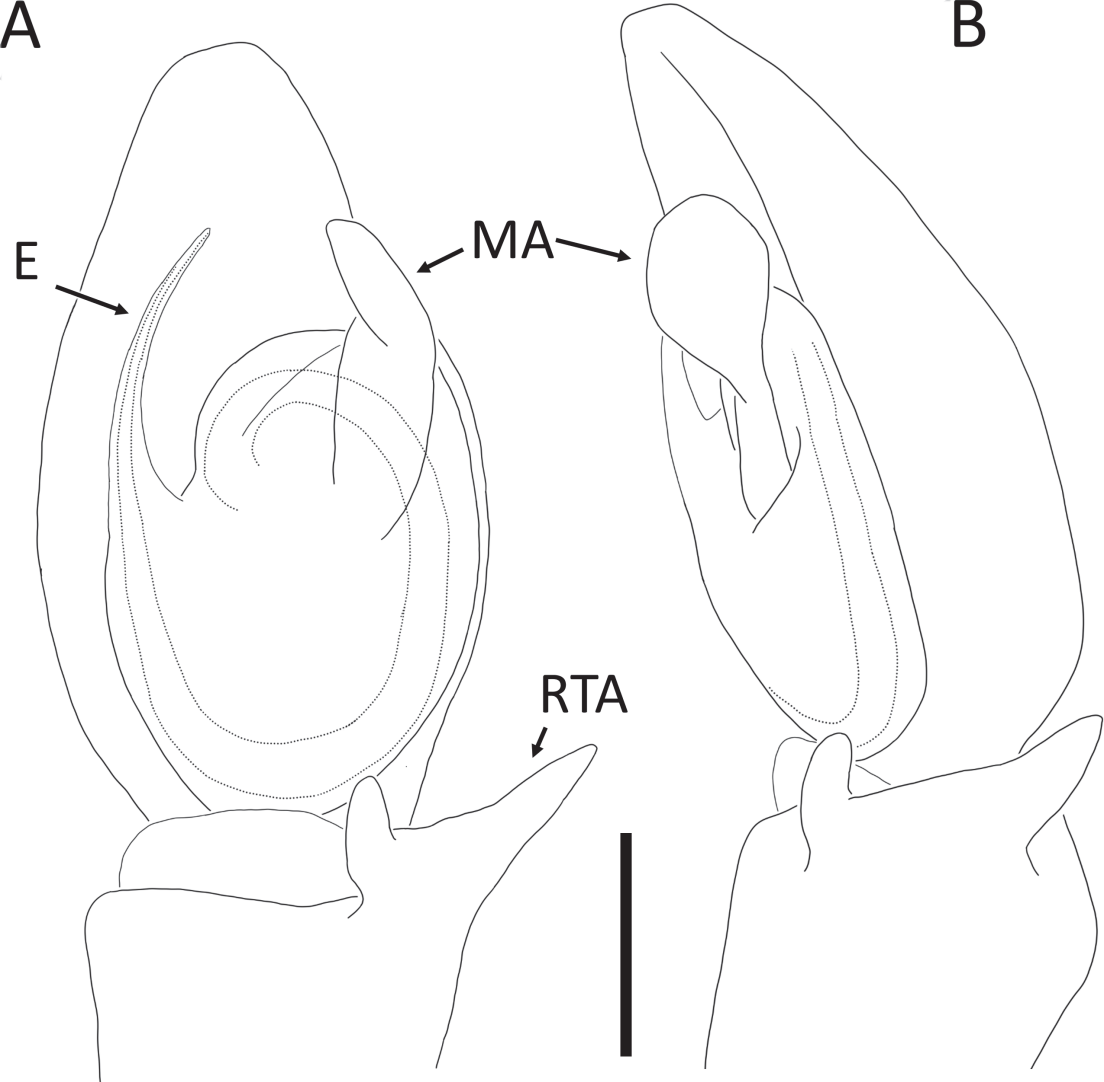
*Angaeusverrucosus* Benjamin, 2017, male (2013_HF3.2_AraThom093N_001) **A, B** left Palp **A** ventral view **B** retrolateral view. Abbreviations: MA = median apophysis; E = embolus; RTA = retrolateral tibial apophysis. Scale bars: 0.2 mm.

**Male** (2013_HF3.2_AraThom093N_001, Figs 3, 4). Total length 5.71. Carapace length 2.80; width 2.70. Abdomen length 2.91; width 2.47. Diameter of eyes: AME 0.09; ALE 0.19; PLE 0.20; PME 0.10. Interdistances between eyes: AME–AME 0.13; AME–ALE 0.12; ALE–ALE 0.52; PME–PME 0.21; PME–PLE 0.26; ALE–PLE 0.23; AME–PME 0.35; PLE–PLE 0.90. Leg measurements: leg I 14.04 (4.32, 1.20, 4.47, 2.78, 1.27); leg II 13.87 (4.29, 0.98, 4.59, 2.80, 1.21); leg III 14.95 (1.93, 9.66, 1.42, 1.19, 0.75); leg IV 6.85 (2.39, 0.62, 1.63, 1.40, 0.81). For a complete description, see Benjamin (2017a).

#### Distribution.

Indonesia (Sumatra: Jambi Province, new record); Malaysia (Borneo) Fig. 29.

### ﻿*Cebrenninus* Simon, 1887

#### ﻿ Cebrenninus magnus

Taxon classificationAnimaliaAraneaeThomisidae

Benjamin, 2016

56552CC7-0CB0-5E81-8340-DF6F3B63912A

Figs 5 , 6

Cebrenninus rugosus Simon, 1887: Tang and Li 2010: 23, figs 17A–C, 18A–E, 19A–D; Benjamin 2011: 13, figs 5C, F, 8B, E, F, 27A–E, 28A–F, 29A–F [misidentifications].
Cebrenninus magnus Benjamin, 2016: 185, figs 6–8, 35–36, 38–45.

##### Material examined.

Indonesia – Sumatra, Jambi Province • 1♂; Sarolangun, Air Hitam, Desa Baru; 02°01'49.5"S, 102°46'14.8"E; elev. 57 m; 22 Jun. 2013; J. Drescher leg.; canopy fogging in jungle rubber plantation; GOET 2013_BJ6.1_AraThom070N_001 (to be transferred to MZB).

##### Diagnosis.

See Benjamin (2016).

##### Description.

**Figure 5.**
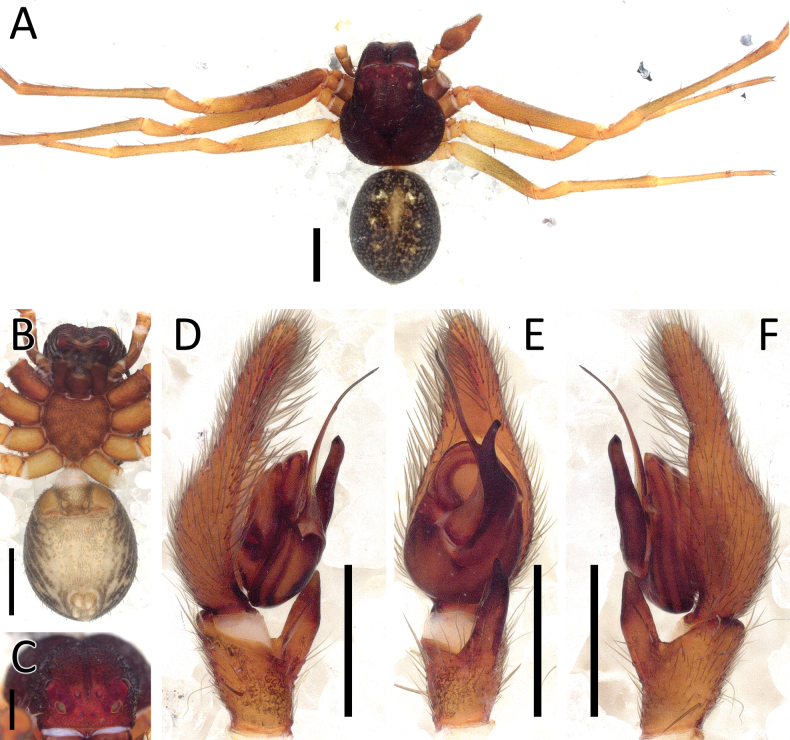
*Cebrenninusmagnus* Benjamin, 2016, male (2013_BJ6.1_AraThom070N_001) **A, B** habitus **A** dorsal view **B** ventral view **C** eye region, frontal view **D–F** left Palp **D** prolateral view **E** ventral view **F** retrolateral view. Scale bars: 1 mm (**A, B**); 0.5 mm (**C**); 0.2 mm (**D–F**).

**Figure 6.**
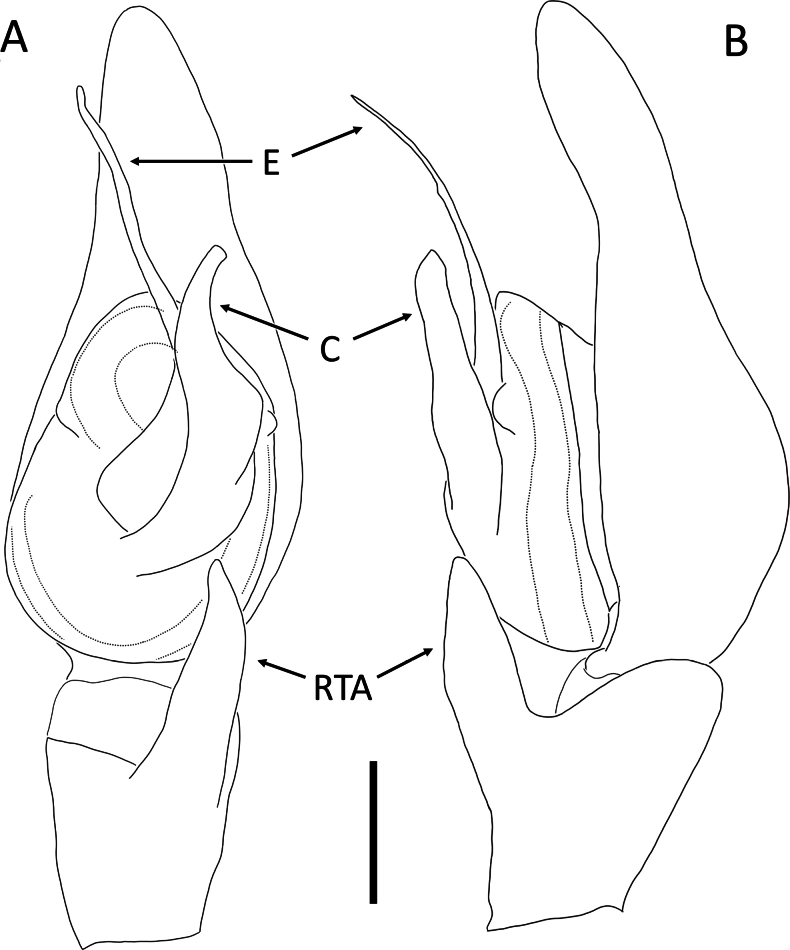
*Cebrenninusmagnus* Benjamin, 2016, male (2013_BJ6.1_AraThom070N_001) **A, B** left Palp **A** ventral view **B** retrolateral view. Abbreviations: C = conductor; E = embolus; RTA = retrolateral tibial apophysis. Scale bars: 0.2 mm.

**Male** (2013_BJ6.1_AraThom070N_001; Figs 5, 6). Total length 4.28. Carapace length 2.06; width 1.99. Abdomen length 2.22; width 1.74. Diameter of eyes: AME 0.06; ALE 0.18; PLE 0.14; PME 0.07. Interdistances between eyes: AME–AME 0.16; AME–ALE 0.18; ALE–ALE 0.58; PME–PME 0.22; PME–PLE 0.33; ALE–PLE 0.20; AME–PME 0.21; PLE–PLE 0.85. Leg measurements: leg I 8.42 (2.56, 0.60, 2.79, 1.91, 0.56); leg II 9.48 (2.76, 0.61, 2.97, 2.03, 1.11); leg III 6.10 (1.80, 0.50, 1.62, 1.41, 0.77); leg IV 6.20 (1.75, 0.52, 1.67, 1.46, 0.80). For complete description, see Benjamin (2011) and Tang and Li (2010) under *Cebrenninusrugosus* Simon, 1887.

##### Distribution.

China; Laos; Thailand; Indonesia (Sumatra: Jambi Province, new record; Java); Malaysia (Borneo) Fig. 29.

#### ﻿ Cebrenninus rugosus

Taxon classificationAnimaliaAraneaeThomisidae

Simon, 1887

6D1F8020-BE98-54CC-A06A-5AE859F599C4

Figs 7 , 8

Cebrenninus rugosus Simon, 1887 468; Simon 1897: 9, figs 1, 2; Benjamin 2016: 190, figs 9, 11, 12, 17–20, 37, 57–64.
Cebrenninus annulatus Simon, 1897: 8.
Cebrenninus scabriculus Simon, 1897: 8.
Libania annulata Thorell, 1890: 149.
Libania armillata Thorell, 1890: 149.
Libania scabricula Thorell, 1890: 148.
Libania scabricula sulcata Thorell, 1890: 148.

##### Material examined.

Indonesia • Sumatra, Jambi Province, 1♀; Sarolangun, Air Hitam, Desa Baru; 02°01'49.5"S, 102°46'14.8"E; elev. 57 m; 12 Jul. 2013; J. Drescher leg.; canopy fogging in jungle rubber plantation; GOET 2013_BJ6.1_AraThom048N_001 (to be transferred to MZB).

##### Diagnosis.

For diagnosis of *Cebrenniusrugosus* Simon, 1887 see Benjamin (2016).

##### Description.

**Figure 7.**
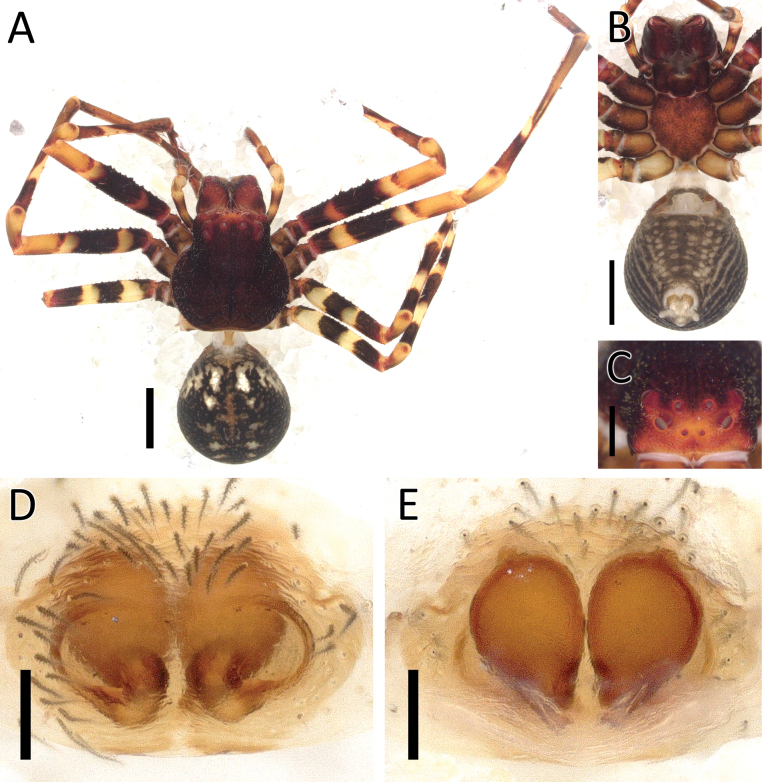
*Cebrenninusrugosus* Simon, 1887, female (2013_BJ6.1_AraThom048N_001) **A, B** habitus **A** dorsal view **B** ventral view **C** eye region, frontal view **D** left femur I, prolateral view. Scale bars: 1 mm (**A, B**); 0.5 mm (**C**); 0.1 mm (**D, E**).

**Figure 8.**
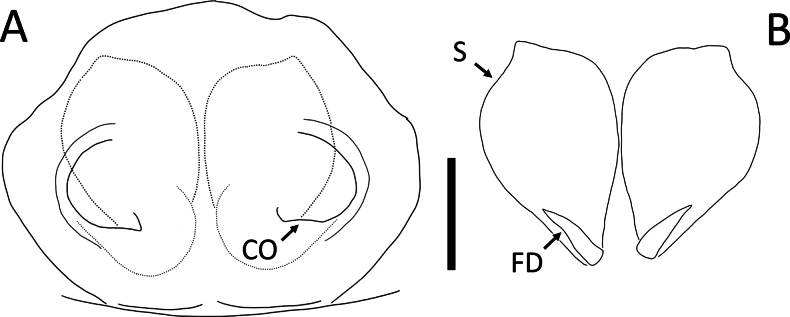
*Cebrenninusrugosus* Simon, 1887, female (2013_BJ6.1_AraThom048N_001) **A, B** epigynum **A** ventral view **B** dorsal view. Abbreviations: CO = copulatory opening; FD = fertilisation ducts; S = spermatheca. Scale bar: 0.1 mm.

**Female** (2013_BJ6.1_AraThom048N_001; Figs 7, 8). Total length 4.35. Carapace length 2.08; width 2.05. Abdomen length 2.27; width 1.95. Diameter of eyes: AME 0.06; ALE 0.13; PLE 0.15; PME 0.07. Interdistances between eyes: AME–AME 0.14; AME–ALE 0.16; ALE–ALE 0.57; PME–PME 0.25; PME–PLE 0.29; ALE–PLE 0.23; AME–PME 0.24; PLE–PLE 0.85. Leg measurements: leg I 8.46 (2.67, 0.62, 2.63, 1.61, 0.93); leg II 8.68 (2.74, 0.68, 2.69, 1.61, 0.96); leg III 5.43 (1.63, 0.47, 1.52, 1.18, 0.63); leg IV 5.73 (1.77, 0.46, 1.54, 1.28, 0.68). For a complete description of *C.rugosus* see Benjamin (2016). The specimen described here possesses striated legs with granulation on the ventral side of femora (Fig. 7A, D). Epigynum (Fig. 8): CO positioned at the posterior end of spermatheca, connected to a semicircular window. Spermatheca oval with a small protrusion on the anterior end.

##### Distribution.

Malaysia (Borneo); Indonesia (Sumatra, Java, Borneo) Fig. 29.

##### Remarks.

The specimen examined here is tentatively identified as *Cebrenninusrugosus*, as no images are available showing the dorsal view of the epigynum, and we are unable to re-examine any type specimen. Examined specimen also exhibits distinct banding and strong ventral granulation on the legs, which were not described in Benjamin (2016), although the lack of banding might be due to the fade of colouration in ethanol.

### ﻿*Crockeria* Benjamin, 2016

#### ﻿ Crockeria kinabalu

Taxon classificationAnimaliaAraneaeThomisidae

Benjamin, 2016

9D528044-AE5B-5B17-A024-8F674CB01880

Figs 9 , 10

Crockeria kinabalu Benjamin, 2016: 195, figs 92, 93, 96–99.

##### Material examined.

Indonesia – Sumatra, Jambi Province • 1♂; Batang Hari, Bajubang, Bungku; 01°55'41.6"S, 103°15'34.2"E; elev. 48 m; 9 May 2013; J. Drescher leg.; canopy fogging in jungle rubber plantation; GOET 2013_HJ1.1_AraThom033N_001 (to be transferred to MZB).

##### Diagnosis.

**Figure 9.**
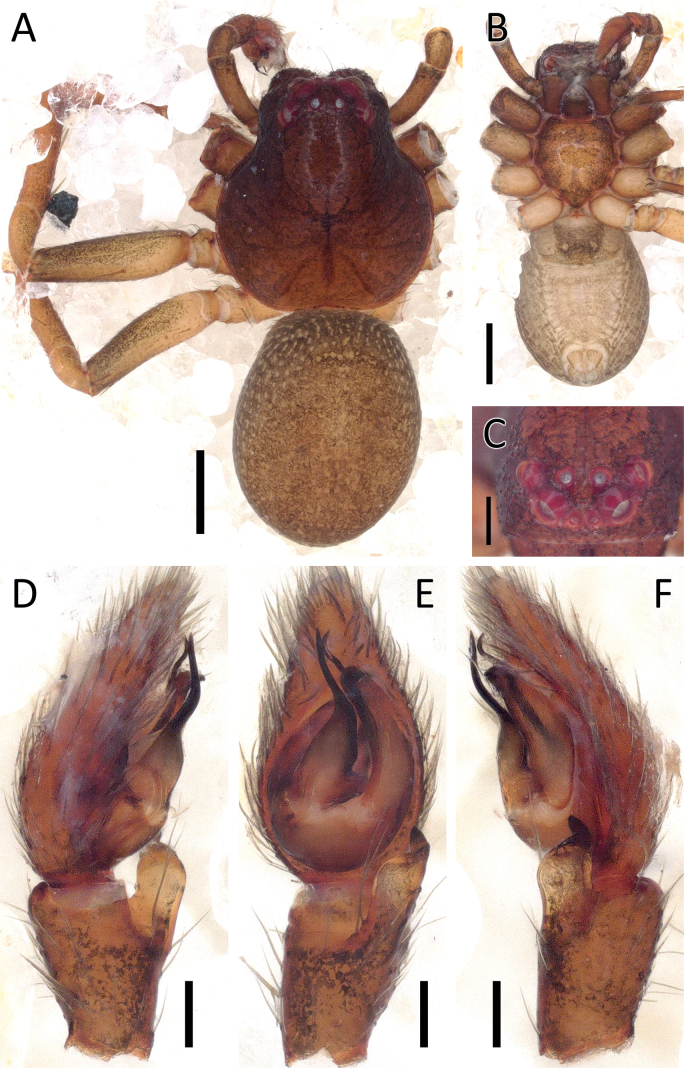
*Crockeriakinabalu* Benjamin, 2016, male (2013_HJ1.1_AraThom033N_001) **A, B** habitus **A** dorsal view **B** ventral view **C** eye region, frontal view **D–F** right palp (mirrored) **D** prolateral view **E** ventral view **F** retrolateral view. Scale bars: 0.5 mm (**A, B**); 0.2 mm (**C**); 0.1 mm (**D–F**).

**Figure 10.**
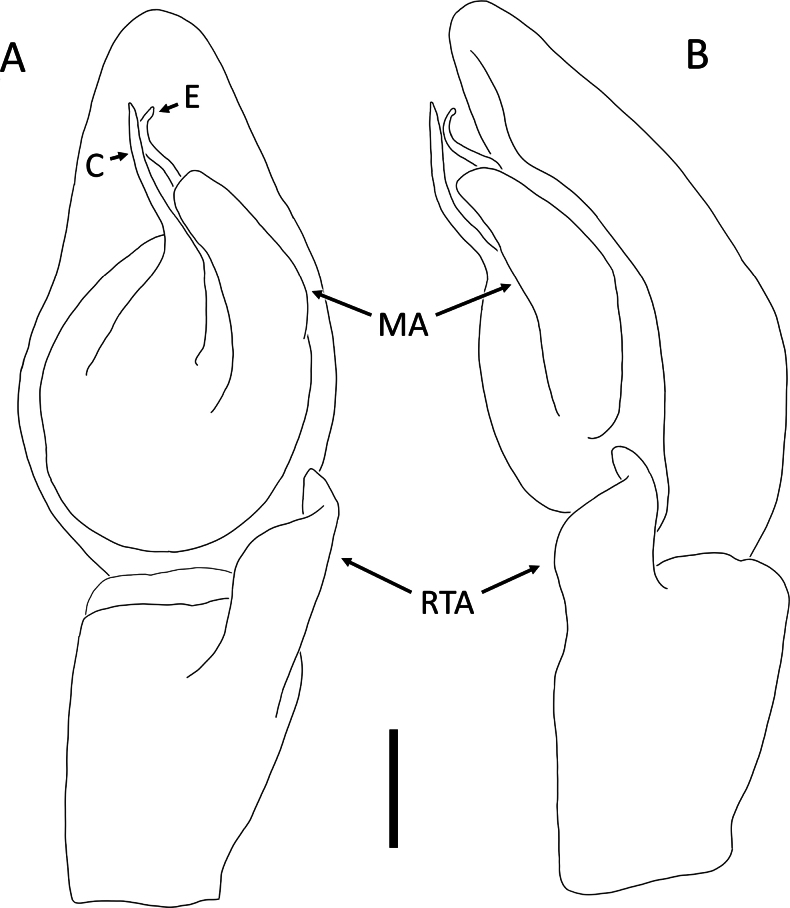
*Crockeriakinabalu* Benjamin, 2016, male (2013_HJ1.1_AraThom033N_001) **A, B** right palp (mirrored) **A** ventral view **B** retrolateral view. Abbreviations: C = conductor; E = embolus; MA = median apophysis; RTA = retrolateral tibial apophysis. Scale bars: 0.1 mm.

**Figure 11.**
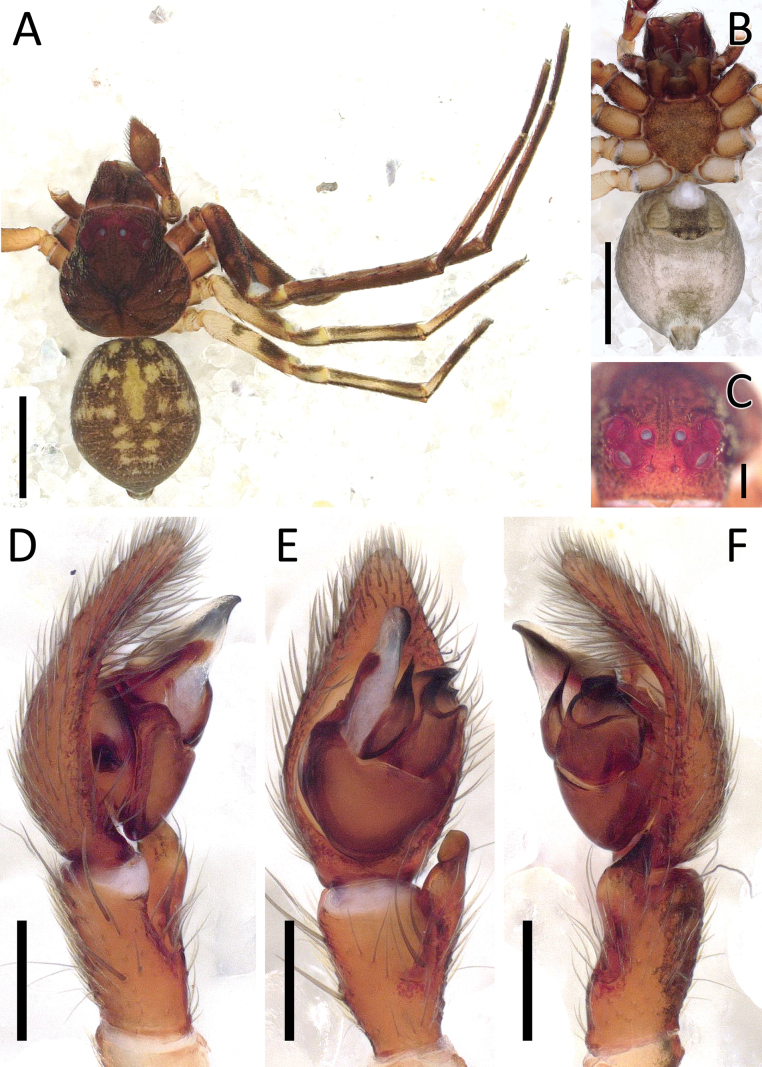
*Crockerianeofelis* Dhiya’ulhaq & Benjamin, sp. nov., male (holotype 2013_BJ5.2_AraThom007N_001) **A, B** habitus **A** dorsal view **B** ventral view **C** eye region, frontal view **D–F** left palp **D** prolateral view **E** ventral view **F** retrolateral view. Scale bars: 1 mm (**A, B**); 0.2 mm (**C–F**).

**Figure 12.**
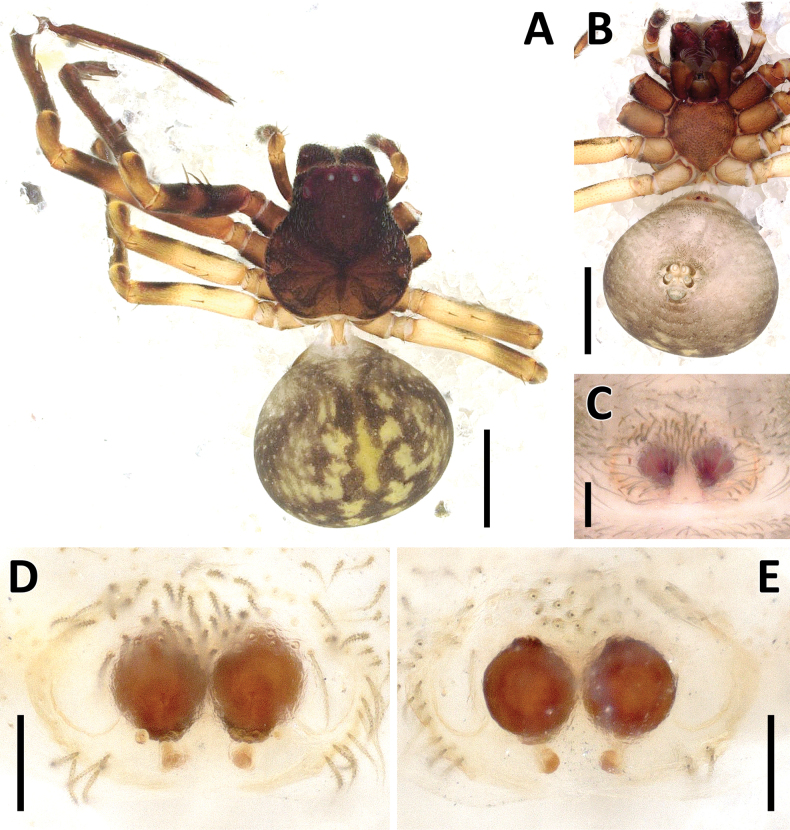
*Crockerianeofelis* Dhiya’ulhaq & Benjamin, sp. nov., female (paratype 2013_BJ5.2_AraThom007N_002) **A, B** habitus **A** dorsal view **B** ventral view **C–E** epigynum **C** ventral view **D** cleared, ventral view **E** cleared, dorsal view. Scale bars: 1 mm (**A, B**); 0.2 mm (**C–E**).

**Figure 13.**
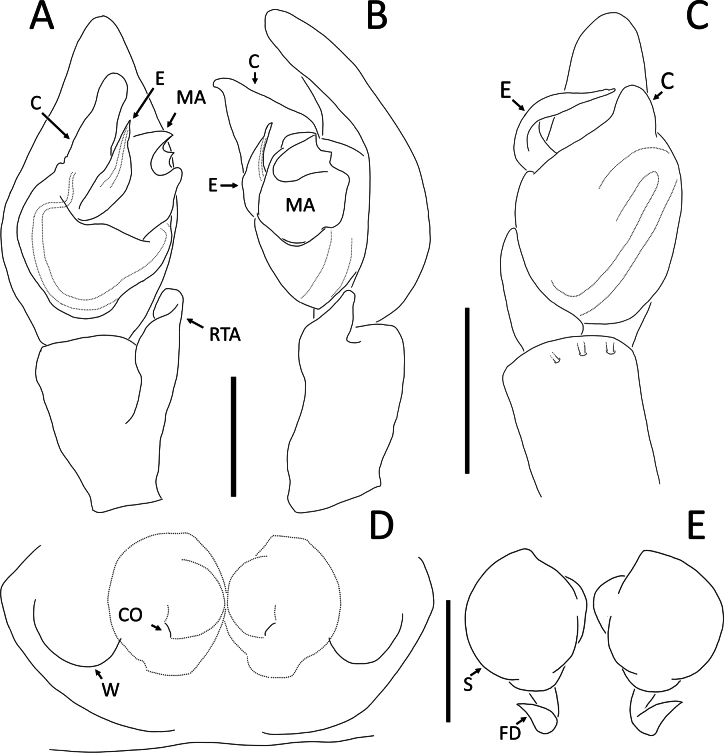
**A, B, D, E***Crockerianeofelis* Dhiya’ulhaq & Benjamin, sp. nov **A, B** male (holotype 2013_BJ5.2_AraThom007N_001), left palp **A** ventral view **B** retrolateral view **D, E** female (paratype 2013_BJ5.2_AraThom007N_002), epigynum **D** ventral view **E** dorsal view **C***Epidiuselongatus* Benjamin, 2017, male (2013_BJ4.1_AraThom071N_001), left palp, ventral view. Abbreviations: C = conductor; CO = copulatory opening; E = embolus; FD = fertilisation duct; MA = median apophysis; RTA = retrolateral tibial apophysis; S = spermatheca; W = epigynal window. Scale bars: 0.2 mm (**A, B**); 0.5 mm (**C**), 0.1 mm (**D, E**).

Males of *Crockeriakinabalu* Benjamin, 2016 are distinguishable from *Crockerianeofelis* Dhiya’ulhaq & Benjamin, sp. nov., by the elongated MA (Figs 9D–F, 10; Benjamin 2016: fig. 96) (vs wide, pincer-shaped; Figs 11E, F, 13A, B); tapering, filiform conductor (vs membranous, triangular with rounded-tip); filiform embolus (vs short, needle-shaped); hook-shaped RTA (Figs 9D, 10A; Benjamin 2016: fig. 97) (vs RTA with a flat, spatulate tip; Figs 11E, 13A). Females are distinguishable from *Crockerialaevis* (Thorell, 1890) by the smaller CO (Benjamin 2016: figs 95, 98, 99) and from *C.neofelis* by the larger, inward-facing CO (vs smaller, outward facing; Figs 12C, D, 13D) and lack of epigynal windows. Furthermore, the abdomen is uniformly coloured (Fig. 9A)(vs patterned in the other two species (Figs 11A, 12A; Benjamin 2016: fig. 94).

##### Description.

**Male** (2013_HJ1.1_AraThom033N_001; Figs 9, 10). Total length 2.75. Carapace length 1.36; width 1.28. Abdomen length 1.39; width 1.14. Diameter of eyes: AME 0.04; ALE 0.12; PLE 0.10; PME 0.05. Interdistances between eyes: AME–AME 0.05; AME–ALE 0.06; ALE–ALE 0.24; PME–PME 0.09; PME–PLE 0.13; ALE–PLE 0.10; AME–PME 0.15; PLE–PLE 0.44. Leg measurements: leg I -; leg II -; leg III 3.22 (0.95, 0.34, 0.83, 0.64, 0.46); leg IV 3.12 (0.96, 0.29, 0.79, 0.62, 0.46). For a complete description see Benjamin (2016).

##### Distribution.

Indonesia (Sumatra: Jambi Province (new record) Fig. 29.

#### ﻿ Crockeria neofelis

Taxon classificationAnimaliaAraneaeThomisidae

Dhiya’ulhaq & Benjamin sp. nov.

9CBD622F-6836-516E-9E0A-50D5AF5F41CF

https://zoobank.org/C649133B-C9E5-4E28-A61E-C4EA7C5F21CF

Figs 11 , 12 , 13A, B, D, E

##### Type material.

***Holotype*.** Indonesia – Jambi Province • 1♂; Sarolangun, Pauh, Semaran; 02°08'35.9"S, 102°51'04.5"E; elev. 45 m; 16 Jul. 2013; J. Drescher leg.; canopy fogging in jungle rubber plantation; GOET 2013_BJ5.2_AraThom007N_001 (to be transferred to MZB). ***Paratypes*.** Indonesia – Jambi Province • 1♀; same data as holotype; GOET 2013_BJ5.2_AraThom007N_002 (to be transferred to MZB). • 2♀♀; Sarolangun, Pauh, Semaran; 02°08'35.9"S, 102°51'04.5"E; elev. 45 m; 16 Jul. 2013; J. Drescher leg.; canopy fogging in jungle rubber plantation; ZMHZMH-A0031849, ZMH-A0031850 (GOET 2013_BJ5.1_AraThom007N_001, 002). • 2♂♂, 1♀; Sarolangun, Air Hitam, Desa Baru; 02°01'49.5"S, 102°46'14.8"E; elev. 57 m; 12 Jul. 2013; J. Drescher leg.; canopy fogging in jungle rubber plantation; ZMHZMH-A0031851, ZMH-A0031852, ZMH-A0031853 (GOET 2013_BJ6.2_AraThom007N_001–003). • 1♀; Sarolangun, Bukit Duabelas National Park; 01°58'55.2"S, 102°45'02.6"E; elev. 73 m; 7 Oct. 2013; J. Drescher leg.; canopy fogging in rainforest; GOET 2013_BF2.1_AraThom007N_001 (to be transferred to SMF).

##### Diagnosis.

Males of *Crockerianeofelis* Dhiya’ulhaq & Benjamin, sp. nov., are easily distinguished from *Crockeriakinabalu* Benjamin, 2016 by the very wide MA with pincer-shaped tip (Figs 11E, F, 13A, B vs elongated with a rounded tip, Figs 9D–F, 10, Benjamin 2016: fig. 96), membranous, triangular conductor with rounded-tip (vs tapering and filiform), short, needle-shaped embolus (vs filiform), and RTA with a flat, spatulate tip (Figs 11E, 13A vs hook-shaped, Figs 9D, 10A, Benjamin 2016: fig. 97). Females are easily distinguished from *C.kinabalu* and *Crockerialaevis* (Thorell, 1890) by the small, outward-facing CO (Fig. 10D, F vs inward facing in *C.kinabalu* and *C.laevis*, Benjamin 2016: figs 95, 98, 99) and the presence of two large, semicircular windows (vs absent in other species). Additionally, the abdomen of both sexes are patterned in a similar way as *C.laevis* (Figs 11A, 12A; Benjamin 2016: fig. 94), unlike the uniformly coloured abdomen in *C.kinabalu* (Fig. 9A).

##### Description.

**Male** (holotype 2013_BJ5.2_AraThom007N_001; Figs 11, 13A, B). Total length 2.91. Carapace length 1.33; width 1.30. Abdomen length 1.58; width 1.25. Diameter of eyes: AME 0.05; ALE 0.14; PLE 0.09; PME 0.07. Interdistances between eyes: AME–AME 0.11; AME–ALE 0.09; ALE–ALE 0.28; PME–PME 0.14; PME–PLE 0.17; ALE–PLE 0.12; AME–PME 0.15; PLE–PLE 0.56. Leg measurements: leg I 6.50 (1.84, 0.46, 1.89, 1.52, 0.79); leg II 6.65 (1.84, 0.44, 1.97, 1.57, 0.83); leg III 3.59 (1.04, 0.32, 0.95, 0.83, 0.45); leg IV 3.63 (1.11, 0.30, 0.97, 0.82, 0.43). Carapace pear-shaped, dark brown, with sparse yellow setae; eye region purplish red; AER recurved; PER recurved. Abdomen oval, brown with paired pale-brown blotches and a pale-brown cardiac mark. Legs with ventral dark blotches and faint striation; front legs dark-brown; back legs pale brown. Palp (Figs 11D–F, 13A, B): cymbium slightly less than twice the length of tibia. Conductor triangular with a rounded tip, membranous, longer than MA and embolus. Embolus short; tip rather stout and needle-shaped. MA very wide, pincer-shaped in ventral view; tip with a sinuous keel. RTA slightly constricted in the middle, followed by a flat, spatulate tip best seen in ventral view.

**Female** (paratype 2013_BJ5.2_AraThom007N_002; Figs 12, 13D, E). Total length 3.42. Carapace length 1.49; width 1.52. Abdomen length 1.93; width 2.02. Diameter of eyes: AME 0.05; ALE 0.12; PLE 0.12; PME 0.07. Interdistances between eyes: AME–AME 0.16; AME–ALE 0.12; ALE–ALE 0.49; PME–PME 0.19; PME–PLE 0.23; ALE–PLE 0.13; AME–PME 0.18; PLE–PLE 0.69. Leg measurements: leg I 6.73 (1.78, 0.46, 2.04, 1.64, 0.81); leg II 6.96 (1.89, 0.47, 2.11, 1.67, 0.82); leg III 3.96 (1.17, 0.38, 1.05, 0.86, 0.50); leg IV 3.95 (1.16, 0.30, 1.05, 0.96, 0.48). Habitus as in males. Epigynum (Figs 12C–F, 13D, E): CO small and inconspicuous, outward facing, positioned at the mid-length of spermatheca. Two semicircular ‘windows’ present beside the CO. CD short. Spermatheca round.

##### Etymology.

The specific epithet refers to the Sunda Clouded Leopard, *Neofelisdiardi* (G. Cuvier, 1823), native to Sumatra. The dark blotches on the legs of *C.neofelis* are reminiscent of the fur pattern of this feline. Noun in apposition.

##### Distribution.

Indonesia (Sumatra: Jambi Province) Fig. 29.

### ﻿*Epidius* Thorell, 1877

#### ﻿ Epidius elongatus

Taxon classificationAnimaliaAraneaeThomisidae

Benjamin, 2017

36985E67-D7DC-5EB4-BDD0-42A4C07000C4

Figs 13C , 14

Epidius elongatus Benjamin, 2017b: 474, figs 2C, 4B.

##### Material examined.

Indonesia – Jambi Province • 1♂; Sarolangun, Air Hitam, Desa Baru; 02°00'56.8"S, 102°45'12.6"E; elev. 64 m; 23 Jun. 2013; J. Drescher leg.; canopy fogging in jungle rubber plantation; GOET 2013_BJ4.1_AraThom071N_001 (to be transferred to MZB).

##### Diagnosis.

See Benjamin (2017b).

##### Description.

**Figure 14.**
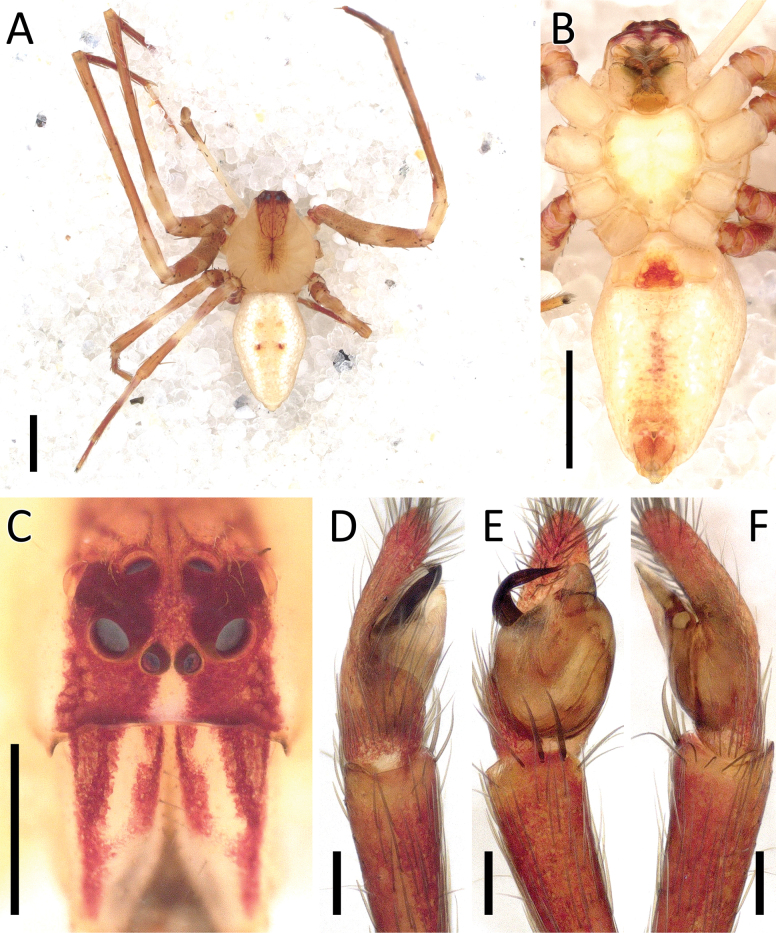
*Epidiuselongatus* Benjamin, 2017, male (2013_BJ4.1_AraThom071N_001) **A, B** habitus **A** dorsal view **B** ventral view **C** eye region, frontal view **D–F** left palp **D** prolateral view **E** ventral view **F** retrolateral view. Scale bars: 1 mm (**A, B**); 0.5 mm (**C**); 0.2 mm (**D–F**).

**Male** (2013_BJ4.1_AraThom071N_001, Figs 13C, 14). Total length 3.95. Carapace length 1.87; width 1.53. Abdomen length 2.08; width 1.31. Diameter of eyes: AME 0.06; ALE 0.14; PLE 0.11; PME 0.08. Interdistances between eyes: AME–AME 0.07; AME–ALE 0.05; ALE–ALE 0.26; PME–PME 0.10; PME–PLE 0.10; ALE–PLE 0.11; AME–PME 0.23; PLE–PLE 0.47. Leg measurements: leg I 11.66 (3.41, 0.62, 3.72, 2.64, 1.27); leg II 11.13 (3.31, 0.62, 3.57, 2.46, 1.17); leg III 5.63 (1.75, 0.41, 1.61, 1.27, 0.59); leg IV 6.21 (1.91, 0.46, 1.81, 1.45, 0.58). Carapace pale yellow, with a reddish brown wide median band running from the cephalic groove across the eyes, splitting just after them, continuing to the chelicerae. Legs faintly striated. Abdomen with a pair of white longitudinal bands. For a complete description, see Benjamin (2017b).

##### Distribution.

Thailand; Indonesia (Sumatra: Jambi Province (new record) Fig. 29.

### ﻿*Ibana* Benjamin, 2014

#### ﻿ Ibana svarnadvipa

Taxon classificationAnimaliaAraneaeThomisidae

Dhiya’ulhaq & Benjamin sp. nov.

63A4A6EA-2E67-518C-9ADB-BC23F114FBC5

https://zoobank.org/A0A2DC07-E41F-4661-9154-391216CA5BFE

Figs 15 , 16 , 17

Epidius rubropictus Benjamin, 2011: 15, figs 5I, 35D, E (female from Sumatra only) [misidentification].

##### Type material.

***Holotype*.** Indonesia – Jambi Province • 1♂; Batang Hari, Bajubang, Sungkai; 01°50'58.7"S, 103°18'00.5"E; elev. 56 m; 5 Jun. 2013; J. Drescher leg.; canopy fogging in jungle rubber plantation; GOET 2013_HJ3.2_AraThom035N_001 (to be transferred to MZB). ***Paratype*.** Indonesia – Jambi Province • 1♂; same data as holotype; ZMHZMH-A0031854 (GOET 2013_HJ3.2_AraThom035N_002). • 1♀; Sarolangun, Air Hitam, Lubuk Kepayang; 02°05'06.8"S, 102°47'20.9"E; elev. 70 m; 23 Jun. 2013; J. Drescher leg.; canopy fogging in rubber plantation; GOET 2013_BR2.2_AraThom035N_001 (to be transferred to MZB).

##### Diagnosis.

**Figure 15.**
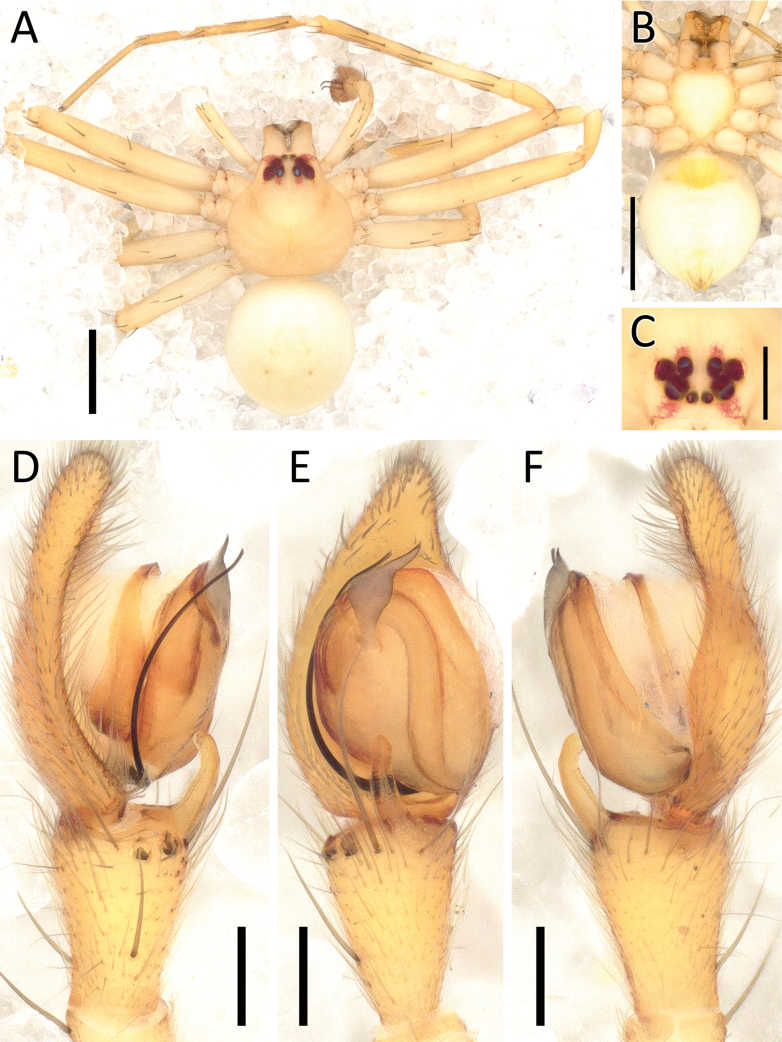
*Ibanasvarnadvipa* Dhiya’ulhaq & Benjamin, sp. nov., male (holotype 2013_HJ3.2_AraThom035N_001) **A, B** habitus **A** dorsal view **B** ventral view **C** eye region, frontal view **D–F** left palp **D** prolateral view **E** ventral view **F** retrolateral view. Scale bars: 1 mm (**A, B**); 0.5 mm (**C**); 0.2 mm (**D–F**).

**Figure 16.**
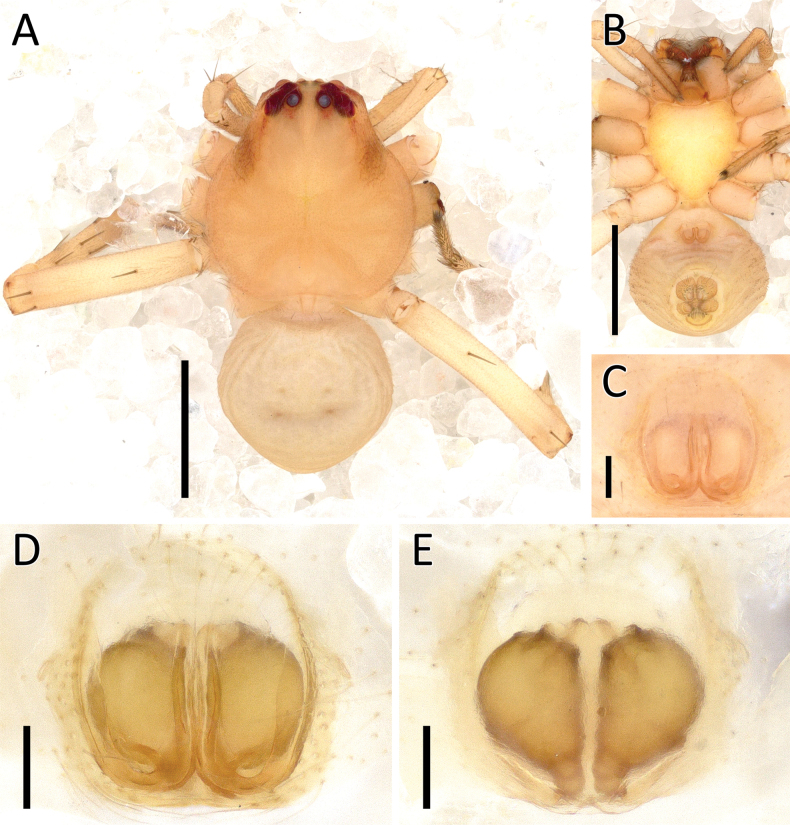
*Ibanasvarnadvipa* Dhiya’ulhaq & Benjamin, sp. nov., female (paratype 2013_BR2.2_AraThom035N_001) **A, B** habitus **A** dorsal view **B** ventral view **C–E** epigynum **C** ventral view **D** cleared, ventral view **E** cleared, dorsal view. Scale bars: 1 mm (**A, B**); 0.1 mm (**C–E**).

**Figure 17.**
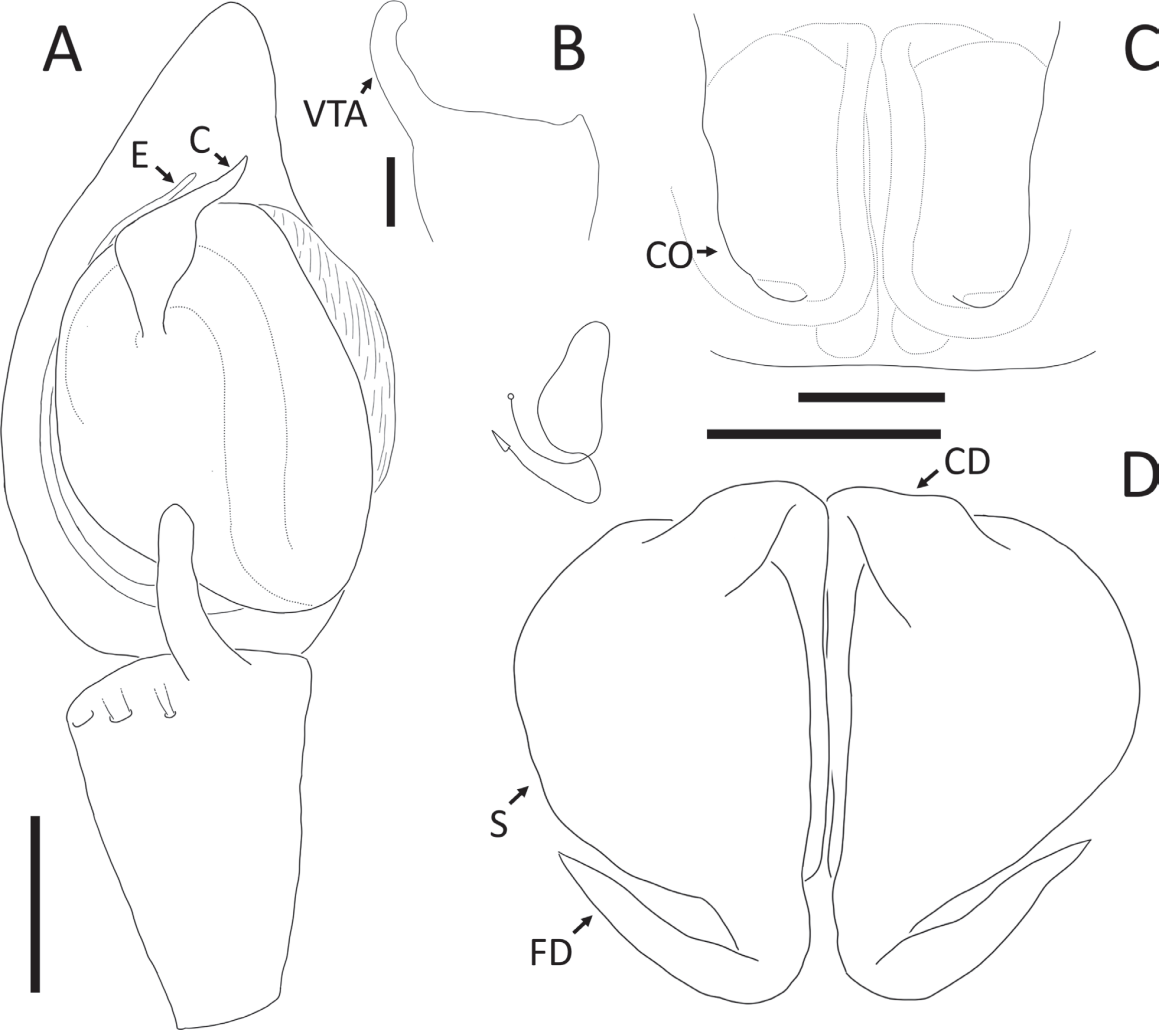
*Ibanasvarnadvipa* Dhiya’ulhaq & Benjamin, sp. nov **A, B** male (holotype 2013_HJ3.2_AraThom035N_001), left palp **A** ventral view **B** tibia, retrolateral view **C, D** female (paratype 013_BR2.2_AraThom035N_001), epigynum **C** ventral view **D** dorsal view. Abbreviations: C = conductor; CD = copulatory duct; CO = copulatory opening; E = embolus; FD = fertilisation duct; MA = median apophysis; S = spermatheca; VTA = ventral tibial apophysis. Scale bars: 0.2 mm (A); 0.1 mm (**B–D**).

Males of *Ibanasvarnadvipa* Dhiya’ulhaq & Benjamin, sp. nov., can be distinguished from *Ibanagan* Liu & S. Q. Li, 2022 and *Ibanasenagang* Benjamin, 2014 by the slightly dorsally curved VTA (Figs 15D, F, 17B vs oblique with a flexed-back tip in *I.senagang*, Benjamin 2016: fig. 3A; absent in *I.gan*, Zhong, Zheng & Liu, 2022: fig. 1E–G) and roughly triangular conductor with long-tapering tip (Figs 15E, 17A vs triangular with abruptly narrowing tip in *I.senagang*; absent in *I.gan*). Females can be distinguished by the CO positioned at the posterior end of spermatheca (Fig. 17C vs anterior to spermatheca in *I.gan*, at middle length of spermatheca in *I.senagang*Liu et al. 2022: figs 2, 3; Benjamin 2016: fig. 3B, C). Additionally, both sexes are rather uniformly coloured yellowish brown, with a pair of red stripes that cross the eye region (Figs 15A–C, 16A, B vs abdomen with a large, dark median band *I.gan*, red cephalic stripes absent in both *I.gan* and *I.senagang*; Zhong, Zheng & Liu, 2022: figs 1A, B, 2A, B; Benjamin 2016: fig. 1G).

##### Description.

**Male** (holotype 2013_HJ3.2_AraThom035N_001; Figs 15, 17A). Total length 3.54. Carapace length 1.56; width 1.51. Abdomen length 1.98; width 1.56. Diameter of eyes: AME 0.06; ALE 0.11; PLE 0.12; PME 0.09. Interdistances between eyes: AME–AME 0.07; AME–ALE 0.04; ALE–ALE 0.28; PME–PME 0.14; PME–PLE 0.12; ALE–PLE 0.11; AME–PME 0.19; PLE–PLE 0.53. Leg measurements: leg I 8.55 (2.21, 0.55, 2.45, 2.12, 1.22); leg II 8.95 (2.48, 0.54, 2.59, 2.10, 1.24); leg III 4.55 (1.38, 0.38, 1.26, 1.00, 0.53); leg IV 4.78 (1.47, 0.37, 1.33, 1.12, 0.49). Carapace pear shaped, yellow; cephalic region with a pair of diagonal red stripes that cross the ocular area; AER recurved; PER slightly recurved. Legs yellow, uniformly coloured. Abdomen oval, pale yellow. Palp (Figs 15D–F, 17A, B): cymbium 1.5 × the length of tibia. Conductor subtriangular; distal part tapering. Embolus long, filiform. Tibia with three strong macrosetae, prolateral to the VTA. VTA slightly dorsally curved; tip rounded.

**Female** (paratype 2013_BR2.2_AraThom035N_001; Figs 16, 17C, D). Total length 3.14. Carapace length 1.52; width 1.52. Abdomen length 1.62; width 1.26. Diameter of eyes: AME 0.05; ALE 0.11; PLE 0.12; PME 0.08. Interdistances between eyes: AME–AME 0.08; AME–ALE 0.05; ALE–ALE 0.29; PME–PME 0.15; PME–PLE 0.13; ALE–PLE 0.11; AME–PME 0.19; PLE–PLE 0.55. Leg measurements: leg I 7.72 (2.13, 0.58, 2.28, 1.78, 0.95); leg II 7.86 (2.27, 0.57, 2.28, 1.71, 1.03); leg III 4.08 (1.27, 0.42, 1.05, 0.86, 0.48); leg IV 4.18 (1.39, 0.38, 1.13, 0.94, 0.34). Female habitus as in male, except the carapace and abdomen is slightly darker in colour. Epigynum (Figs 16C–E, 17C, D): atrium longer than wide, posterior half bordered by a thin flap. CO hidden by aforementioned flap, positioned posterior to spermatheca. CD long, going around the inner side of spermatheca before joining at the anterior side. Spermatheca inverted pear-shaped. FD half of spermatheca length.

##### Etymology.

The specific epithet is taken from an ancient name for Sumatra, which is Sanskrit for ‘island of gold’. Also referring to the colouration of the species in ethanol. Noun in apposition.

##### Distribution.

Indonesia (Sumatra: North Sumatra and Jambi Province) Fig. 29.

### ﻿*Pharta* Thorell, 1891

#### ﻿ Pharta bimaculata

Taxon classificationAnimaliaAraneaeThomisidae

Thorell, 1891

0BD7857D-DC87-5F81-92FA-4D94DF37DE02

Figs 18 , 19 , 20

Pharta bimaculata Thorell, 1891: 85; Benjamin 2011: 18, figs 5E, 48A–D.
Sanmenia kohi Ono, 1995: 162, figs 7–15.

##### Material examined.

Indonesia – Jambi Province • 1♂, 1♀; Sarolangun, Air Hitam, Lubuk Kepayang; 02°04'15.2"S, 102°47'30.8"E; elev. 54 m; 5 Jun. 2013; J. Drescher leg.; canopy fogging in oil palm plantation; GOET 2013_BO3.2_AraThom067N_002–003 (to be transferred to MZB). • 1♂; same data as previous; ZMHZMH-A0031855 (GOET 2013_BO3.2_AraThom067N_001). 1♀; Muaro Jambi, Bahar Utara, Talang Bukit; 01°52'41.8"S, 103°21'21.6"E; elev. 21 m; 17 Aug. 2013; J. Drescher leg.; canopy fogging in oil palm plantation; ZMHZMH-A0031856 (GOET 2013_HOr2.2_AraThom067N_001). • 1♀; Batang Hari, Bajubang, Sungkai; 01°51'39.4"S, 103°18'19.0"E; elev. 42 m; 19 Aug. 2013; J. Drescher leg.; canopy fogging in oil palm plantation; GOET 2013_HOr3.1_AraThom067N_001 (to be transferred to SMF).

##### Description.

**Figure 18.**
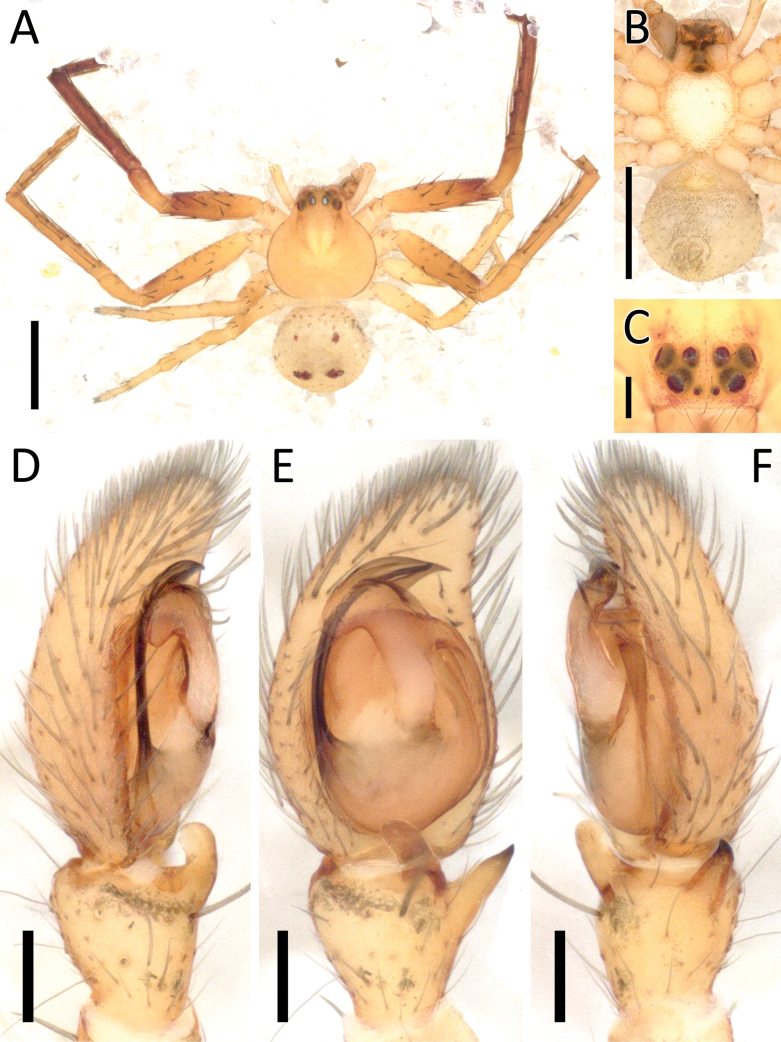
*Phartabimaculata* Thorell, 1891, male (2013_BO3.2_AraThom067N_001) **A, B** habitus **A** dorsal view **B** ventral view **C** eye region, frontal view **D–F** left palp **D** prolateral view **E** ventral view **F** retrolateral view. Scale bars: 1 mm (**A, B**); 0.2 mm (**C**); 0.1 mm (**D–F**).

**Male** (2013_BO3.2_AraThom067N_001; Figs 18, 20A–C). Total length 2.54. Carapace length 1.25; width 1.25. Abdomen length 1.29; width 1.10. Diameter of eyes: AME 0.04; ALE 0.09; PLE 0.09; PME 0.07. Interdistances between eyes: AME–AME 0.07; AME–ALE 0.05; ALE–ALE 0.22; PME–PME 0.09; PME–PLE 0.11; ALE–PLE 0.10; AME–PME 0.13; PLE–PLE 0.43. Leg measurements: leg I 5.30 (1.46, 0.48, 1.52, 1.20, 0.64); leg II 4.95 (1.39, 0.42, 1.46, 1.14, 0.54); leg III 2.64 (0.79, 0.31, 0.69, 0.50, 0.35); leg IV 2.86 (0.88, 0.23, 0.77, 0.61, 0.37). Carapace pear-shaped, yellow, with a thin, black border; cephalic region with a pair of faint, diagonal, red stripes that cross the eyes; AER recurved; PER recurved. Leg I dark brown; leg II paler than leg I; legs III and IV pale yellow. Abdomen oval, cream coloured with a pair of large red spots in the middle and a smaller pair anteriorly, as well as faint small red dots all over. Palp (Figs 18D–F, 20A–C) as described in Ono (1995) under *Sanmeniakohi*.

**Figure 19.**
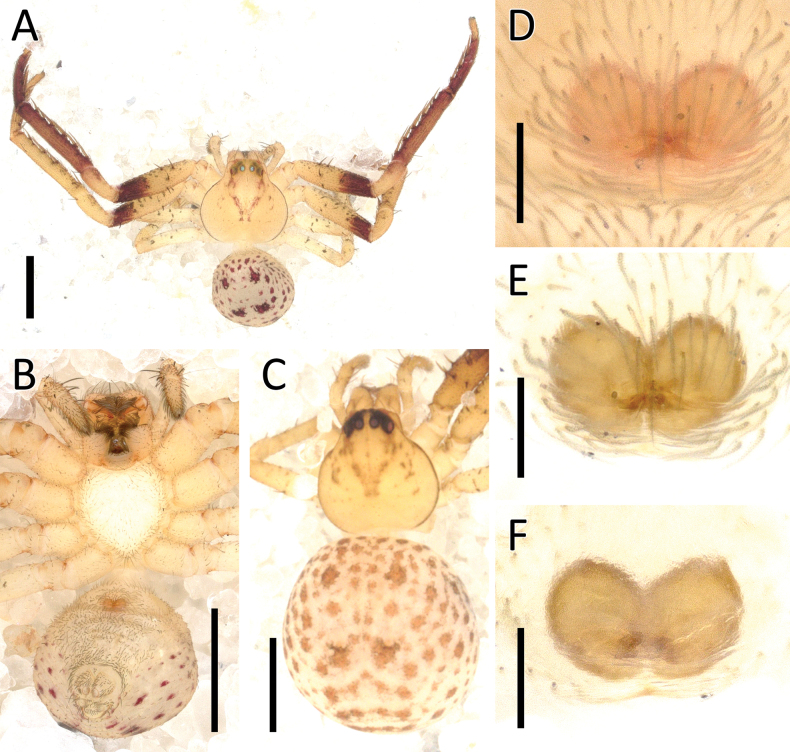
*Phartabimaculata* Thorell, 1891 **A, B, D–F** female (2013_BO3.2_AraThom067N_003) **A, B** habitus **A** dorsal view **B** ventral view **D–F** epigynum **D** ventral view **E** cleared, ventral view **E** cleared, dorsal view **C** female (2013_HJ4.2_AraThom067N_001), habitus, dorsal view. Scale bars: 1 mm (**A–C**); 0.1 mm (**D–F**).

**Figure 20.**
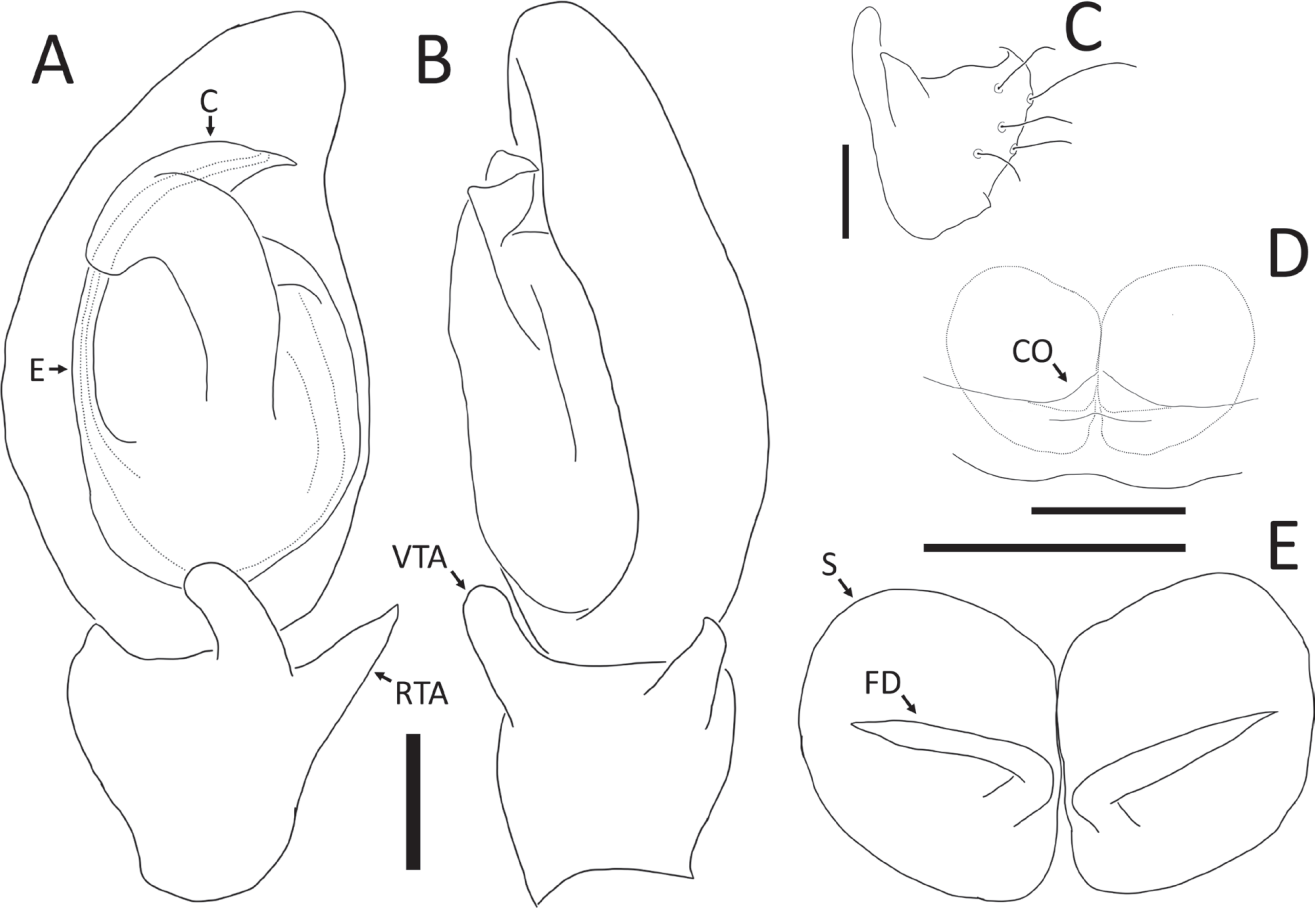
*Phartabimaculata* Thorell, 1891 **A–C** male (2013_BO3.2_AraThom067N_001), left palp **A** ventral view **B** retrolateral view **C** palpal tibia, retrolateral view **D, E** female (2013_BO3.2_AraThom067N_003), epigynum **D** ventral view **E** dorsal view. Abbreviations: C = conductor; CO = copulatory opening; E = embolus; FD = fertilisation duct; MA = median apophysis; RTA = retrolateral tibial apophysis; S = spermatheca; VTA = ventral tibial apophysis. Scale bars: 0.1 mm.

**Female** (2013_BO3.2_AraThom067N_003, Figs 19, 20D, E). Total length 2.82. Carapace length 1.39; width 1.45. Abdomen length 1.43; width 1.30. Diameter of eyes: AME 0.04; ALE 0.11; PLE 0.11; PME 0.07. Interdistances between eyes: AME–AME 0.08; AME–ALE 0.05; ALE–ALE 0.25; PME–PME 0.10; PME–PLE 0.11; ALE–PLE 0.11; AME–PME 0.15; PLE–PLE 0.46. Leg measurements: leg I 5.33 (1.53, 0.59, 1.61, 1.14, 0.46); leg II 5.18 (1.56, 0.53, 1.55, 1.08, 0.46); leg III 2.67 (0.82, 0.31, 0.68, 0.48, 0.38); leg IV 3.03 (0.94, 0.31, 0.82, 0.54, 0.42). Habitus as in male, except the following: red carapace markings are more prominent and reach the middle of carapace. Small red dots on the abdomen are much more prominent. Epigynum: CO positioned roughly in the middle of spermatheca, on the inner margin, connected to a long horizontal fold. Spermatheca oval.

##### Distribution.

Singapore; Indonesia (Sumatra: Jambi Province; new record) Fig. 29.

##### Variation.

Certain specimens have less vibrant markings on the carapace and abdomen (Fig. 19D).

##### Remarks.

The male specimen described here aligns perfectly with the characteristics of the genus, confirming the associated female’s accuracy. However, the genitalia of the female specimen described by Benjamin (2011: fig. 48C, D) does not align with the female described in this study. Namely, it possesses a ‘ring’ encircling the vulva, and the horizontal fold is noticeably lacking. Furthermore, the male and female specimens described by Benjamin (2011) were not collected during the same sampling event, suggesting a possible mismatch between them. Given that this is the type species of the genus and the type specimen is a juvenile, minimising taxonomic confusion is essential. Therefore, the current material will be described as it is, while acknowledging the uncertainties. To resolve this issue definitively, we propose awaiting additional specimens from Singapore, the terra typica. Broader sampling will help clarify the taxonomic placement and relationships, providing a more accurate and comprehensive understanding of this taxon in future.

#### ﻿ Pharta roseomaculata

Taxon classificationAnimaliaAraneaeThomisidae

Dhiya’ulhaq & Benjamin sp. nov.

ED6F2879-4E0C-578D-8627-DB938822013B

https://zoobank.org/A254A6D8-9815-44D1-9664-924A2359B9F4

Figs 21 , 22

##### Type material.

***Holotype*.** Indonesia – Jambi Province • 1♀; Batang Hari, Hutan Harapan Conservation Area; 02°09'09.3"S, 103°21'41.8"E; elev. 65 m; 19 Jul. 2013; J. Drescher leg.; canopy fogging in rainforest; GOET 2013_HF1.1_AraThom044N_001 (to be transferred to MZB).

##### Diagnosis.

**Figure 21.**
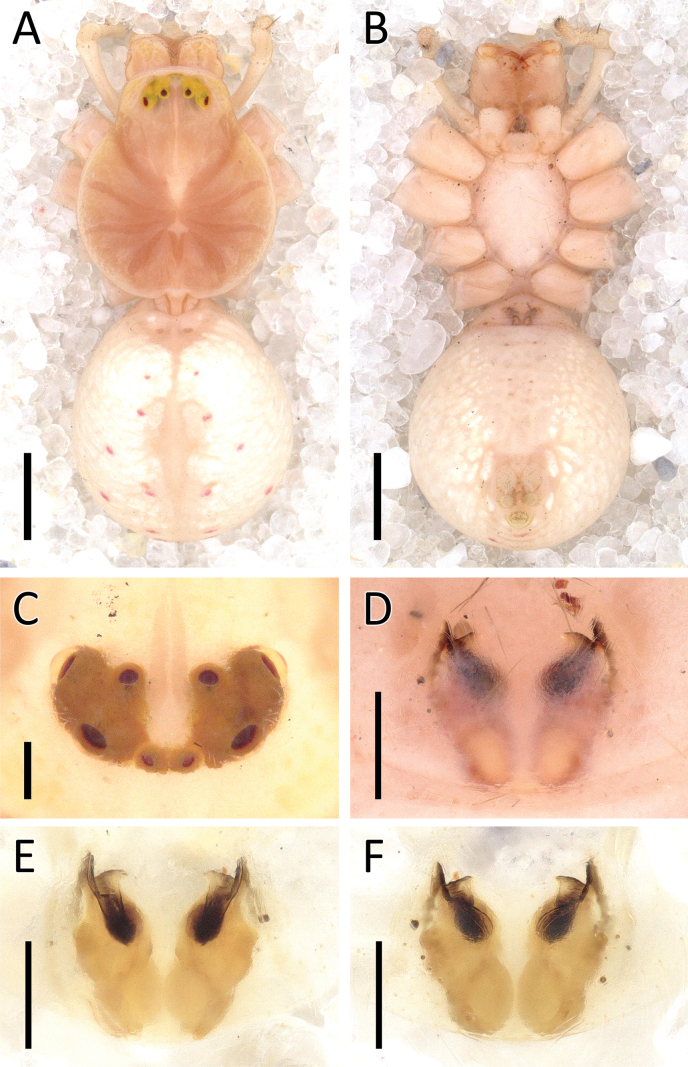
*Phartaroseomaculata* Dhiya’ulhaq & Benjamin sp. nov., female (holotype 2013_HF1.1_AraThom044N_001) **A, B** habitus **A** dorsal view **B** ventral view **C** eye region, frontal view **D–F** epigynum **D** ventral view **E** cleared, ventral view **F** cleared, dorsal view. Scale bars: 1 mm (**A, B**); 0.2 mm (**C–F**).

**Figure 22.**
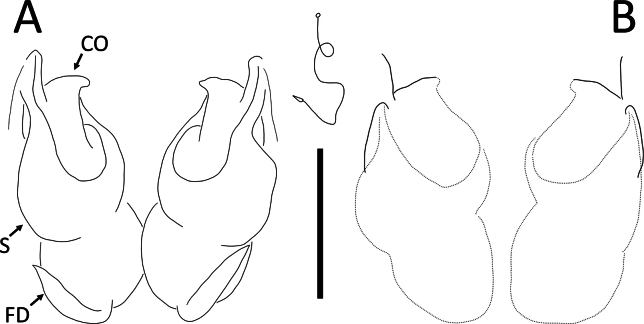
*Phartaroseomaculata* Dhiya’ulhaq & Benjamin sp. nov., female (holotype 2013_HF1.1_AraThom044N_001) **A, B** epigynum **A** dorsal view **B** ventral view. Abbreviations: CO = copulatory opening; FD = fertilisation duct; S = spermatheca. Scale bars: 0.2 mm.

Females of *Phartaroseomaculata* Dhiya’ulhaq & Benjamin, sp. nov., are similar to *Phartasudmannorum* Benjamin, 2014 in having a roughly 8-shaped spermatheca and very dark, funnel-shaped CO, but can be distinguished by outward facing CO (Figs 21D–F, 22 vs inward facing, Benjamin 2014: figs 1E, F, 2E, C) and the anterior part of the spermatheca is slightly larger than the posterior part (vs posterior part larger than anterior).

##### Description.

**Female** (holotype 2013_HF1.1_AraThom044N_001, Figs 21, 22). Total length 5.81. Carapace length 2.50; width 2.35. Abdomen length 3.31; width 2.63. Diameter of eyes: AME 0.05; ALE 0.12; PLE 0.12; PME 0.07. Interdistances between eyes: AME–AME 0.10; AME–ALE 0.13; ALE–ALE 0.44; PME–PME 0.21; PME–PLE 0.17; ALE–PLE 0.17; AME–PME 0.26; PLE–PLE 0.64. Leg measurements: leg I 12.09 (3.67, 1.01, 3.74, 2.52, 1.15); leg II -; leg III 6.05 (1.94, 0.67, 1.67, 1.18, 0.59); leg IV 6.97 (2.28, 0.67, 2.16, 1.17, 0.69). Carapace pear-shaped, pale-pink coloured; eye region yellow; AER recurved; PER recurved. Legs uniformly pale pink. Abdomen oval, dorsally with seven pairs of small pink spots.

Epigynum (Figs 21D–F, 22): CO semi-oval. CD funnel-shaped, anteriorly attached to a sclerotised ‘handle’, ending in a half-loop, very dark in comparison to spermatheca. Spermatheca 8-shaped; anterior part slightly larger than posterior part.

##### Etymology.

The specific epithet is taken from Latin and refers to the pink spots on the abdomen.

##### Distribution.

Indonesia (Sumatra: Jambi Province) Fig. 29.

#### ﻿ Rangkayo

Taxon classificationAnimaliaAraneaeThomisidae

Dhiya’ulhaq & Benjamin gen. nov.

4CCC8EE2-1079-5E35-B1DF-0B084B45CF9C

https://zoobank.org/7FD186DF-D786-4DF4-A4B5-DAE94158656B

##### Type species.

*Rangkayohitam* Dhiya’ulhaq & Benjamin, sp. nov.

##### Diagnosis.

Among the Oriental thomisids of the *Epidius* clade (sensu Benjamin 2011), *Rangkayo* Dhiya’ulhaq & Benjamin, gen. nov., is similar to *Epidius* Thorell, 1877 and *Ibana* Benjamin, 2014 in possessing a row of strong macrosetae ventrally on the distal edge of male palpal tibia and the lack of MA, but can be distinguished from both genera by the shape of cymbium with a prominent apical protrusion and presence of tegular bump. Additionally, it is distinguished from *Epidius* by the long, whiplike embolus and tibia shorter than cymbium and from *Ibana* by the larger conductor with a wide base. The tibia of the two described species lack TA, a feature also absent in some species of the other two genera (*Ibanagan* Liu & S. Q. Li, 2022; *Epidiusmahavira* Benjamin, 2017b; *Epidiuslongimanus* Benjamin, 2017b). Females are unique in having a large, butterfly shaped atrium; very long, convoluted CD arranged in loops, turns, and folds; small, inconspicuous spermatheca. The latter two characters appear instead superficially similar to the dietine genus *Lycopus* Thorell, 1895.

##### Description.

Total body length 3–4 mm. Carapace pear-shaped, slightly longer than wide. PLE>ALE>PME>AME. AER recurved, PER slightly recurved. Legs long and slender; front legs much longer than back legs; leg formula 2143; tarsi and tibiae I and II with several pairs of ventral spines. Abdomen oval. Male palp: cymbium elongated, with an apical extension; embolus very long, whip-like, arising from an extension of the tegulum; conductor present; tegular bump present, situated next to the conductor; tibia ventrally with a row of macrosetae. Epigynum: Atrium large, butterfly-shaped; copulatory ducts very long and convoluted, consisting of loops, turns, and fold; spermatheca small and inconspicuous. Colouration: whole body yellow; eye region slightly darker; abdomen with several pairs of red and white spots dorsally. Hardly any somatic difference between sexes.

##### Etymology.

The generic name, as well as the type species, is taken from Rangkayo Hitam or Orang Kayo Hitam, a legendary king of Jambi central to the foundation myth of Jambi City. Gender masculine.

##### Species composition.

*Rangkayohitam* Dhiya’ulhaq & Benjamin, sp. nov., *Rangkayoperkaso* Dhiya’ulhaq & Benjamin, sp. nov.

#### ﻿ Rangkayo hitam

Taxon classificationAnimaliaAraneaeThomisidae

Dhiya’ulhaq & Benjamin sp. nov.

ED513DFB-C6E2-5FCF-AC32-CD183EE69BCA

https://zoobank.org/52E30870-0EA8-4040-B586-222A4E860634

Figs 23 , 24 , 25

##### Type material.

***Holotype*.** Indonesia – Jambi Province • 1♂; Batang Hari, Hutan Harapan Conservation Area; 02°09'52.9"S, 103°22'04.0"E; elev. 51 m; 2 Aug. 2013; J. Drescher leg.; canopy fogging in rainforest; GOET 2013_HFr1.2_AraThom091N_001 (to be transferred to MZB). ***Paratype*.** Indonesia – Jambi Province • 1♀; Batang Hari, Bajubang, Singkawang; 01°47'07.9"S, 103°16'37.4"E; elev. 56 m; 18 Jun. 2013; J. Drescher leg.; canopy fogging in jungle rubber plantation; GOET 2013_HJ4.1_AraThom091N_001 (to be transferred to MZB). • 1♀; Batang Hari, Bajubang, Pompa Air; 01°49'33.3"S, 103°17'38.1"E; elev. 51 m; 14 May 2013; J. Drescher leg.; canopy fogging in jungle rubber plantation; GOET 2013_HJ2.1_AraThom091N_001 (to be transferred to MZB).

##### Diagnosis.

**Figure 23.**
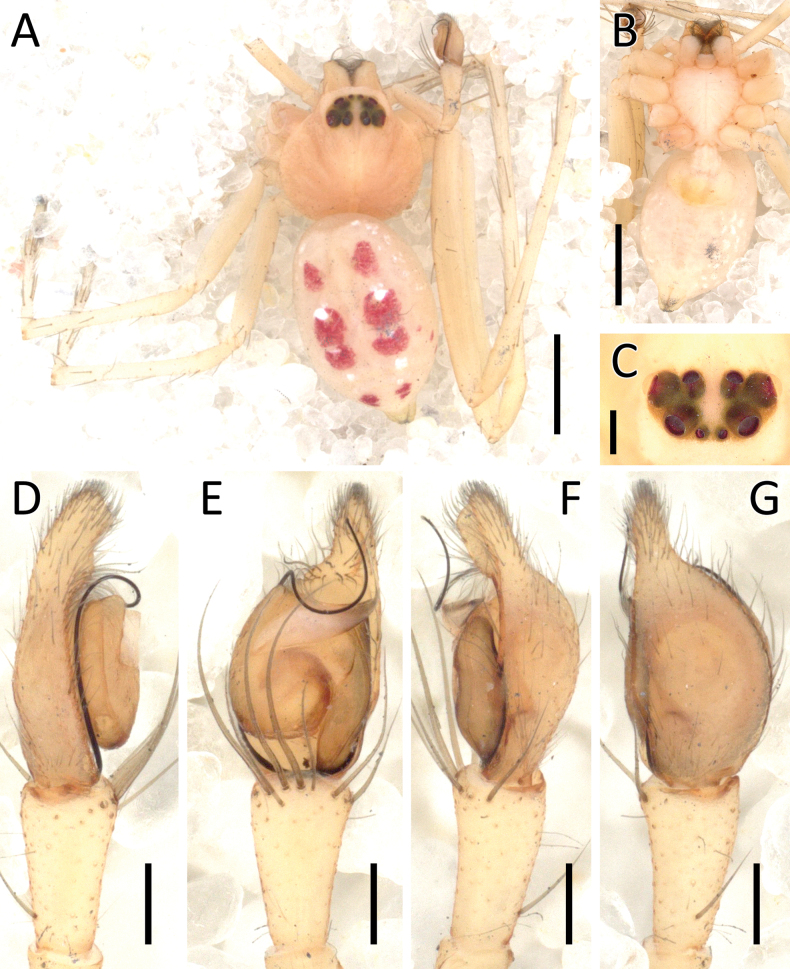
*Rangkayohitam* Dhiya’ulhaq & Benjamin, sp. nov., male (holotype 2013_HFr1.2_AraThom091N_001) **A, B** habitus **A** dorsal view **B** ventral view **C** eye region, frontal view **D–G** right palp (mirrored) **D** prolateral view **E** ventral view **F** retrolateral view **G** dorsal view. Scale bars: 1 mm (**A, B**); 0.2 mm (**C–G**).

**Figure 24.**
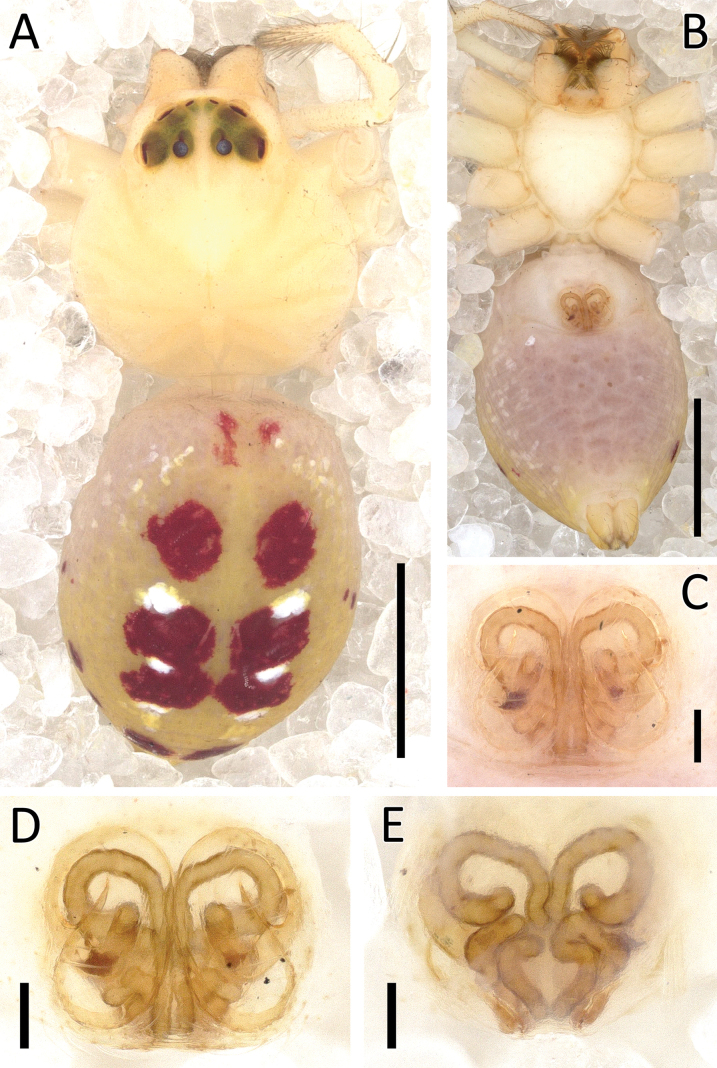
*Rangkayohitam* Dhiya’ulhaq & Benjamin, sp. nov., female (paratype 2013_HJ4.1_AraThom091N_001) **A, B** habitus **A** dorsal view **B** ventral view **C–E** epigynum **C** ventral view **D** cleared, ventral view **E** cleared, dorsal view. Scale bars: 1 mm (**A, B**); 0.1 mm (**C–E**).

**Figure 25.**
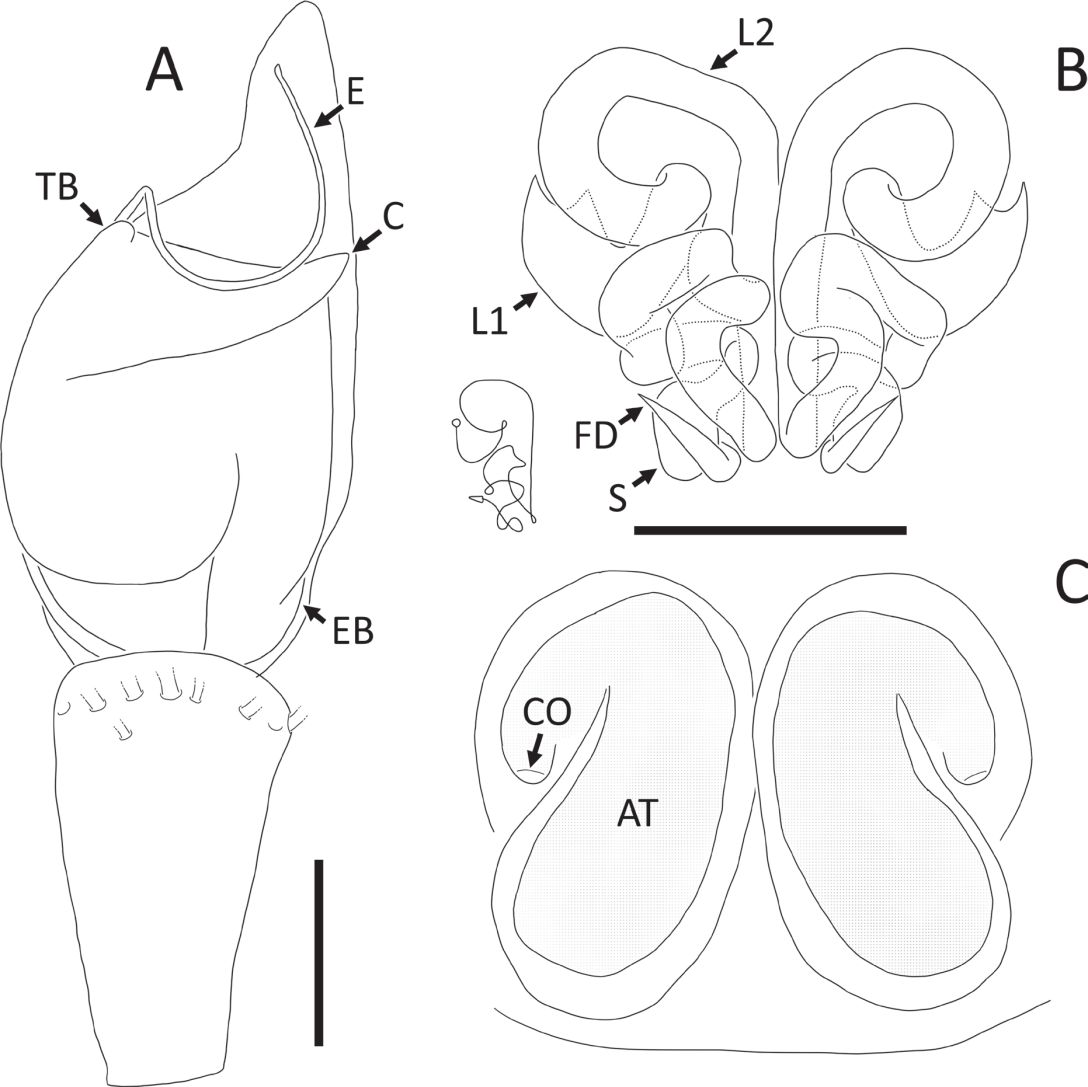
*Rangkayohitam* Dhiya’ulhaq & Benjamin, sp. nov. **A, B** male (holotype 2013_HFr1.2_AraThom091N_001), right palp (mirrored) **A** ventral view **B, C** female (paratype 2013_HJ4.1_AraThom091N_001), epigynum **B** ventral view **C** dorsal view. Abbreviations: AT = atrium; C = conductor; CD = copulatory duct; CO = copulatory opening; E = embolus; EB = embolic base; FD = fertilisation duct; L1 = first loop of copulatory ducts; L2 = second loop of copulatory ducts; MA = median apophysis; S = spermatheca; TB = tegular bump. Scale bars: 0.2 mm.

**Figure 26.**
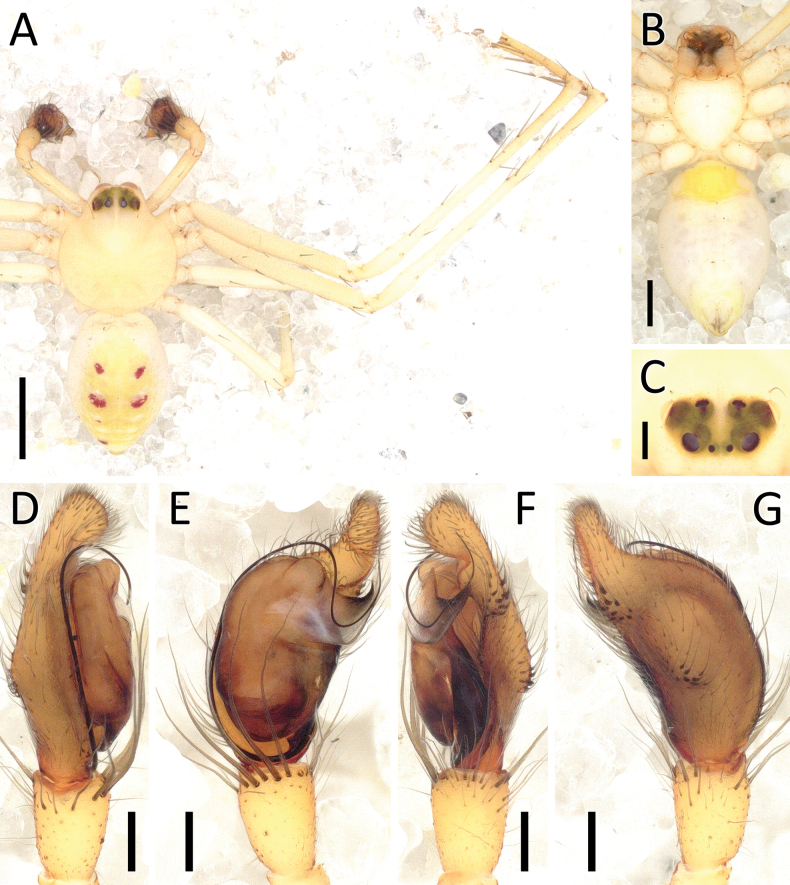
*Rangkayoperkaso* Dhiya’ulhaq & Benjamin, sp. nov., male (holotype 2013_BJ3.2_AraThom056N_001) **A, B** habitus **A** dorsal view **B** ventral view **C** eye region, frontal view **D–G** left palp **D** prolateral view **E** ventral view **F** retrolateral view **G** dorsalview. Scale bars: 1 mm (**A, B**); 0.2 mm (**C–G**).

**Figure 27.**
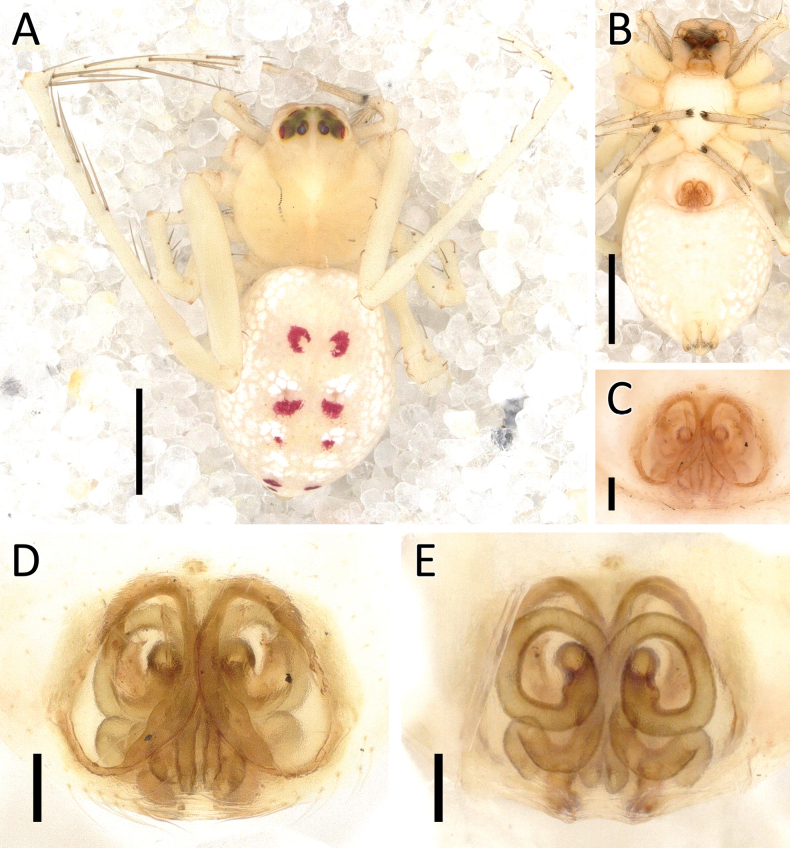
*Rangkayoperkaso* Dhiya’ulhaq & Benjamin, sp. nov., female (paratype 2013_BF4.2_AraThom056N_001) **A, B** habitus **A** dorsal view **B** ventral view **C–E** epigynum **C** ventral view **D** cleared, ventral view **E** cleared, dorsal view. Scale bars: 1 mm (**A, B**); 0.1 mm (**C–F**).

**Figure 28.**
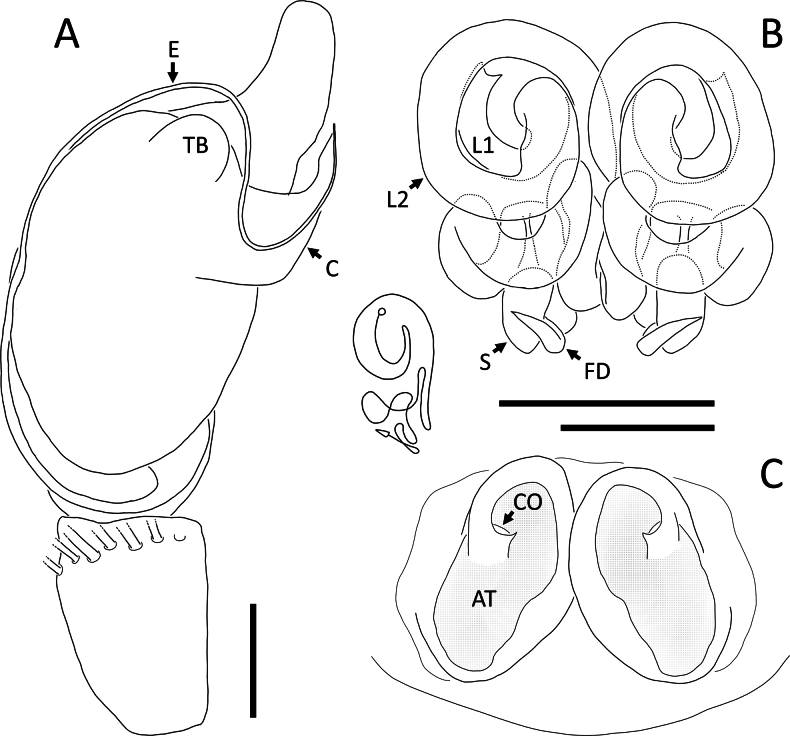
*Rangkayoperkaso* Dhiya’ulhaq & Benjamin, sp. nov. **A, B** male (holotype 2013_BJ3.2_AraThom056N_001), left palp **A** ventral view **B, C** female (paratype 2013_BF4.2_AraThom056N_001), epigynum **B** ventral view **C** dorsal view. Abbreviations: AT = atrium; C = conductor; CD = copulatory duct; CO = copulatory opening; E = embolus; FD = fertilisation duct; L1 = first loop of copulatory ducts; L2 = second loop of copulatory ducts; MA = median apophysis; S = spermatheca; TB = tegular bump. Scale bars: 0.2 mm.

Males of *Rangkayohitam* Dhiya’ulhaq & Benjamin, sp. nov. can be distinguished from the only other species *Rangkayoperkaso* Dhiya’ulhaq & Benjamin, sp. nov. by the straighter cymbium (Fig. 23G vs bent, Fig. 26G), lack of dorsal cymbial setae with widened base (vs present), smaller, round tegulum, not covering most of the embolic base (Figs 23D, 25A vs larger, oval-shaped, covering most of the embolic base, Figs 26D, 28A), longer and straighter conductor (vs shorter and curved), and small tegular bump (vs large). Females can be distinguished by the more spaced CO (Figs 24C, E, 25C), anterior region of atrium as wide as posterior region (vs anterior region narrower than posterior; Figs 27C, E, 28C), CD with a narrower second loop, which does not encircle the first loop (Figs 24D, 25B vs fully encircling the first loop, Figs 27B, 28B), posterior part of CD forming a longitudinal bow (vs transverse bow). Additionally, the abdominal red spots appear much larger and with a deeper colour than in *R.perkaso* (Figs 23A, 24A, 26A, 27A), although this may reflect the condition of the specimens rather than a diagnostic trait.

##### Description.

**Male** (holotype 2013_HFr1.2_AraThom091N_001; Figs 23, 25A). Total length 3.42. Carapace length 1.61; width 1.41. Abdomen length 1.81; width 1.39. Diameter of eyes: AME 0.04; ALE 0.10; PLE 0.12; PME 0.08. Interdistances between eyes: AME–AME 0.07; AME–ALE 0.05; ALE–ALE 0.26; PME–PME 0.12; PME–PLE 0.10; ALE–PLE 0.12; AME–PME 0.22; PLE–PLE 0.51. Leg measurements: leg I 10.25 (2.78, 0.54, 3.19, 2.68, 1.06); leg II 10.62 (2.82, 0.48, 3.40, 2.82, 1.10); leg III 4.57 (1.40, 0.39, 1.33, 0.96, 0.49); leg IV 4.97 (1.51, 0.40, 1.45, 1.07, 0.54). Carapace pear-shaped, yellowish orange; eye region slightly darkened; AER recurved; PER slightly recurved. Legs uniformly yellow. Abdomen oval, yellow; dorsally with four pairs of large, red spots, interspersed by smaller white ones; laterally with a few small red spots in the posterior half. Palp (Figs 23D–G, 25A): Cymbium 1.5 × length of tibia, straight, oval-shaped with a narrowed anterior extension. Tegulum round, not covering the long embolic base. Tegular bump small, prolaterally positioned. Conductor long and rather straight, tapering into a rounded tip. Embolus long, whip-like, curving around the prolateral side of the bulb, before distally curving to the other direction. Tibia ventrally with seven strong macrosetae.

**Female** (paratype 2013_HJ4.1_AraThom091N_001; Figs 24, 25B, C). Total length 3.76. Carapace length 1.60; width 1.48. Abdomen length 2.16; width 1.53. Diameter of eyes: AME 0.05; ALE 0.11; PLE 0.13; PME 0.09. Interdistances between eyes: AME–AME 0.09; AME–ALE 0.06; ALE–ALE 0.28; PME–PME 0.13; PME–PLE 0.13; ALE–PLE 0.13; AME–PME 0.21; PLE–PLE 0.55. Leg measurements: leg I 9.76 (2.66, 0.55, 3.14, 2.43, 0.98); leg II 10.08 (2.83, 0.56, 3.27, 2.52, 0.90); leg III -; leg IV -. Female habitus as in male, except the carapace is paler in colour. Epigynum (Figs 24C–E, 25B, C): Atrium large, butterfly-shaped; anterior region as wide as posterior region. CO semicircular. CD very long and convoluted, anteriorly with two major loops; first loop after the CO appearing only as a half-circle, not encircled by the second loop. Next to the second loop is a straight tube running to the posterior. Posterior part of CD initiated by a longitudinal bow, followed by a series of folds and turns, ending in a rather inconspicuous, oval spermatheca.

##### Etymology.

See under etymology of genus. The specific epithet is a noun in apposition.

##### Distribution.

**Figure 29.**
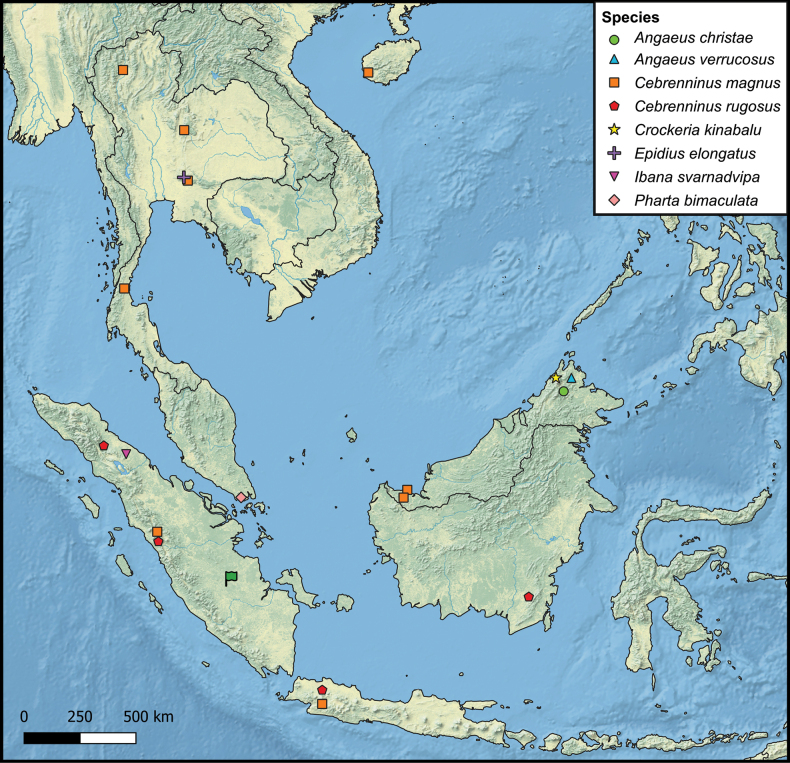
Distribution of examined Thomisidae species from the *Epidius* clade (sensu Benjamin, 2011) prior to the present study, as well as the sampling location of the current study in Jambi Province, Sumatra, Indonesia (green flag). The new genus and the five new species described in the present study are all from the same study location, i.e., a replicated plot design across lowland rainforest, ‘jungle rubber’ (rubber agroforestry), and smallholder monoculture plantations of rubber and oil palm in Jambi’s lowlands (Drescher et al. 2016).

Indonesia (Sumatra: Jambi Province) Fig. 29.

#### ﻿ Rangkayo perkaso

Taxon classificationAnimaliaAraneaeThomisidae

Dhiya’ulhaq & Benjamin sp. nov.

F9E83854-29F4-5831-9F60-9BB31A2CCB12

https://zoobank.org/4900DCA9-B129-406F-BCED-8859FD72D7BB

Figs 26 , 27 , 28

##### Type material.

***Holotype*.** Indonesia – Jambi Province • 1♂; Sarolangun, Air Hitam, Lubuk Kepayang; 02°03'46.6"S, 102°48'03.5"E; elev. 74 m; 26 Jun. 2013; J. Drescher leg.; canopy fogging in jungle rubber plantation; GOET 2013_BJ3.2_AraThom056N_001 (to be transferred to MZB). ***Paratypes*.** Indonesia – Jambi Province • 1♀; Sarolangun, Bukit Duabelas National Park; 01°56'30.8"S, 102°34'50.6"E; elev. 91 m; 4 Oct. 2013; J. Drescher leg.; canopy fogging in rainforest; GOET 2013_BF4.2_AraThom056N_001 (to be transferred to MZB). • 1♂; Sarolangun, Air Hitam, Desa Baru; 02°01'49.5"S, 102°46'14.8"E; elev. 57 m; 12 Jul. 2013; J. Drescher leg.; canopy fogging in jungle rubber plantation; GOET 2013_BJ6.1_AraThom056N_001 (to be transferred to MZB). • 1♂; Batang Hari, Hutan Harapan Conservation Area; 02°09'09.3"S, 103°21'41.8"E; elev. 65 m; 19 Jul. 2013; J. Drescher leg.; canopy fogging in rainforest; ZMHZMH-A0031858 (GOET 2013_HF1.1_AraThom056N_001). • 1♂; Batang Hari, Bajubang, Sungkai; 01°50'58.7"S, 103°18'00.5"E; elev. 56 m; 5 Jun. 2013; J. Drescher leg.; canopy fogging in jungle rubber plantation; GOET 2013_HJ3.2_AraThom056N_001 (to be transferred to SMF).

##### Diagnosis.

Males of *Rangkayoperkaso* Dhiya’ulhaq & Benjamin, sp. nov., can be easily distinguished from the only other species *Rangkayohitam* Dhiya’ulhaq & Benjamin, sp. nov., by the conspicuously bent cymbium (Fig. 26G) (vs straight; Fig. 23G), the presence of dorsal cymbial setae with widened base (vs absent), larger, oval shaped tegulum, covering most of embolic base (Figs 26D, 28A vs smaller, round, not covering most of embolic base, Figs 23D, 25A), a shorter, curved conductor (vs longer and straight), and large tegular bump (vs small). Females can be distinguished by the CO positioned closer together (Figs 27C, E, 28C), the anterior region of the atrium narrower than the posterior region (vs both regions similarly wide; Figs 24C, E, 25C). CD with a wider second loop, fully encircling the first loop (Figs 27D, 28B vs first loop being mostly outside the second loop, Figs 24D, 25B), and the posterior part of CD forming a transverse bow (vs a longitudinal bow).

##### Description.

**Male** (holotype 2013_BJ3.2_AraThom056N_001; Figs 26, 28A). Total length 3.29. Carapace length 1.52; width 1.47. Abdomen length 1.77; width 1.25. Diameter of eyes: AME 0.04; ALE 0.10; PLE 0.12; PME 0.08. Interdistances between eyes: AME–AME 0.07; AME–ALE 0.05; ALE–ALE 0.24; PME–PME 0.13; PME–PLE 0.11; ALE–PLE 0.13; AME–PME 0.22; PLE–PLE 0.51. Leg measurements: leg I 9.99 (2.68, 0.52, 3.13, 2.65, 1.01); leg II 10.38 (2.85, 0.56, 3.23, 2.71, 1.03); leg III 4.46 (1.39, 0.34, 1.34, 0.92, 0.47); leg IV 4.93 (1.54, 0.34, 1.47, 1.05, 0.53). Carapace pear-shaped, yellow; eye region slightly darker; AER recurved; PER slightly recurved. Legs uniformly pale yellow. Abdomen oval; dorsally yellow with two full pairs of red spots and two smaller ones only on the left side; paired white spots very faint; laterally pale. Palp (Figs 26D–F, 28A): cymbium more than 2 × longer than tibia, retrolaterally bent, elongated with a narrowed anterior extension; dorsally with a prominent hump in the centre, as well as two fields of setae with widened base. Tegulum large, oval-shaped, covering the embolic base. Tegular bump large, apically positioned. Conductor with a wide base, abruptly narrowed and strongly curved. Embolus long, whip-like, curving around the prolateral side of the bulb, before distally curving to the other direction. Tibia ventrally with seven strong macrosetae.

**Female** (paratype 2013_BF4.2_AraThom056N_001; Figs 27, 28B, C).

Total length 3.83. Carapace length 1.47; width 1.45. Abdomen length 2.36; width 1.73. Diameter of eyes: AME 0.05; ALE 0.11; PLE 0.13; PME 0.08. Interdistances between eyes: AME–AME 0.08; AME–ALE 0.05; ALE–ALE 0.26; PME–PME 0.13; PME–PLE 0.12; ALE–PLE 0.12; AME–PME 0.22; PLE–PLE 0.53. Leg measurements: leg I 7.81 (2.26, 0.39, 2.38, 1.85, 0.93); leg II -; leg III 4.09 (1.21, 0.35, 1.20, 0.84, 0.49); leg IV 4.62 (1.48, 0.34, 1.37, 0.92, 0.51). Female habitus as in male, except for the following: abdomen with four full pairs of red spots, interspersed with prominent white spots; laterally bordered by a thick white band. Epigynum (Figs 27C–E, 28B, C): atrium large, butterfly-shaped; anterior region narrower than the posterior region. CO semicircular. CD very long and convoluted, anteriorly with two major loops; the first loop after the CO almost forms a full loop, completely encircled by the second loop. Next to the second loop is a straight tube running to the posterior. Posterior part of CD initiated by a slightly meandering tube, followed by a transverse bow, then a few folds and turns before ending in a rather inconspicuous, oval shaped spermatheca.

##### Etymology.

The specific epithet is taken from a Jambi Malay word meaning powerful, which is included in the first line of the traditional song from Jambi ‘Orang Kayo Hitam’: *Rang Kayo Hitam*, *gagah perkaso* (translated: Rang Kayo Hitam, mighty and powerful). Also referring to the rather strong bend of the male palp.

##### Distribution.

Indonesia (Sumatra: Jambi Province) Fig. 29.

## ﻿Discussion

This study results from a long-standing research project, EFForTS, conducted around two forest reserves (Bukit Duabelas National Park & Hutan Harapan) in the province of Jambi, Sumatra, Indonesia. In the first of five canopy fogging sampling campaigns in the span of 12 years, approximately 3000 adult spider specimens were collected. Of these, 263 specimens belong to Thomisidae, assigned to 63 morphospecies. With this, Thomisidae were the third most speciose spider family within our canopy fogging collection, only surpassed by Theridiidae (155 morphospecies) and Salticidae (96 morphospecies) (Ramos et al. 2022).

The description of five new species of Thomisidae as well as new locality records for five known species improves our understanding of the biodiversity of crab spiders in the Oriental region. Thomisids are highly camouflaged predators of flower visitors, and some species groups even can adjust colour and patterns to match the colour of their preferred ambush site, on occasion within days (e.g., *Misumena* spp. or *Thomisus* spp.) (Platnick et al. 2020). Thomisids of the genus *Amyciaea* even mimic ants, such as *A.lineatipes* which mimics the weaver ant *Oecophyllasmaragdina* (Platnick et al. 2020).

The species newly described here, as well as the novel genus *Rangkayo*, still have the strong distinction between forelegs and hindlegs like most thomisids, suggesting that their movement patterns, as well as hunting strategies, are similar to those of well-known thomisid species. The discovery of a new genus within the Thomisidae, *Rangkayo* Dhiya’ulhaq & Benjamin, gen. nov., highlights substantial taxonomic knowledge gaps regarding the remarkable biodiversity of the study region. Continued taxonomic analysis of our EFForTS collections, and other sampling efforts, may likely reveal even more undescribed spider genera, possibly even within the Thomisidae. Additionally, molecular genetic data will further aid in understanding the phylogenetic placement of *Rangkayo* Dhiya’ulhaq & Benjamin, gen. nov., within the thomisid phylogeny, especially concerning the morphological similarity of the male palps with those of *Ibana* and *Epidius*.

## Supplementary Material

XML Treatment for Angaeus christae

XML Treatment for Angaeus verrucosus

XML Treatment for Cebrenninus magnus

XML Treatment for Cebrenninus rugosus

XML Treatment for Crockeria kinabalu

XML Treatment for Crockeria neofelis

XML Treatment for Epidius elongatus

XML Treatment for Ibana svarnadvipa

XML Treatment for Pharta bimaculata

XML Treatment for Pharta roseomaculata

XML Treatment for Rangkayo

XML Treatment for Rangkayo hitam

XML Treatment for Rangkayo perkaso

## ﻿Additional information

### Author ORCIDs

Naufal Urfi Dhiya’ulhaq https://orcid.org/0000-0001-6563-4643

Suresh P. Benjamin https://orcid.org/0000-0003-4666-0330

Damayanti Buchori https://orcid.org/0000-0002-2843-0737

Purnama Hidayat https://orcid.org/0000-0001-9507-6275

Stefan Scheu https://orcid.org/0000-0003-4350-9520

Jochen Drescher https://orcid.org/0000-0002-5162-9779

### Data availability

All of the data that support the findings of this study are available in the main text.

## References

[B1] Benjamin SP Dimitrov D Gillespie RG Hormiga G (2008) Family ties: molecular phylogeny of crab spiders (Araneae: Thomisidae). Cladistics 24: 708–722. 10.1111/j.1096-0031.2008.00202.x

[B2] Benjamin SP (2011) Phylogenetics and comparative morphology of crab spiders (Araneae: *Dionycha*, Thomisidae). Zootaxa 3080(1): 1–108. 10.11646/zootaxa.3080.1.1

[B3] Benjamin SP (2013) On the crab spider genus *Angaeus* Thorell, 1881 and its junior synonym *Paraborboropactus* Tang and Li, 2009 (Araneae: Thomisidae). Zootaxa 3635(1): 71–80. 10.11646/zootaxa.3635.1.7 26097932

[B4] Benjamin SP (2014) Two new species of *Pharta* Thorell, 1891 with the description of *Ibanasenagang* gen. et sp. nov. (Araneae: Thomisidae). Zootaxa 3894(1): 177–182. 10.11646/zootaxa.3894.1.15 25544630

[B5] Benjamin SP (2016) Revision of *Cebrenninus* Simon, 1887 with description of one new genus and six new species (Araneae: Thomisidae). Revue Suisse de Zoologie 123(1): 179–200. 10.5281/zenodo.46304

[B6] Benjamin SP (2017a) A new species of *Angaeus* from Malaysia with possible affinity to related genera within Stephanopinae (Araneae: Thomisidae). Zootaxa 4337(2): 297–300. 10.11646/zootaxa.4337.2.10 29242447

[B7] Benjamin SP (2017b) Distributional and taxonomic notes on the crab spider genus *Epidius* with descriptions of five new species (Araneae: Thomisidae). Journal of Natural History 51(9–10): 469–485. 10.1080/00222933.2017.1302016

[B8] Drescher J Rembold K Allen K Beckschäfer P Buchori D Clough Y Faust H Fauzi AM Gunawan D Hertel D Irawan B Jaya INS Klarner B Kleinn C Knohl A Kotowska MM Krashevska V Krishna V Leuschner C Lorenz W Meijide A Melati D Nomura M Pérez-Cruzado C Qaim M Siregar IT Steinebach S Tjoa A Tscharntke T Wick B Wiegand K Kreft H Scheu S (2016) Ecological and socio-economic functions across Tropical Land Use Systems after rainforest conversion. Philosophical Transactions of the Royal Society B: Biological Sciences 371: 20150275. 10.1098/rstb.2015.0275 PMC4843696 27114577

[B9] Kulkarni S Wood HM Hormiga G (2023) Advances in the reconstruction of the spider tree of life: A roadmap for spider systematics and comparative studies. Cladistics 39(6): 479–532. 10.1111/cla.12557 37787157

[B10] Liu KK Li WH Yao YB Li CZ Li SQ (2022) The first record of the thomisid genus *Ibana* Benjamin, 2014 (Araneae, Thomisidae) from China, with the description of a new species. Biodiversity Data Journal 10: e93637. 10.3897/BDJ.10.e93637 PMC9836584 36761648

[B11] Ono H (1995) Four East Asian spiders of the families Eresidae, Araneidae, Thomisidae and Salticidae (Arachnida, Araneae). Bulletin of the National Museum of Nature and Science Tokyo (A) 21: 157–169.

[B12] Platnick NI Hormiga G Jäger P Jocqué R Ramírez MJ Raven RJ (2020) Spiders of the World. A natural history. In Platnick NI (Ed.) Spiders of the World. Princeton University Press. 10.1525/9780691204987

[B13] Pollierer MM Drescher J Potapov A Kasmiatun Mawan A Mutiari M Nazarreta R Hidayat P Buchori D Scheu S (2023) Rainforest conversion to plantations fundamentally alters energy fluxes and functions in canopy arthropod food webs. Ecology Letters 26(10): 1663–1675. 10.1111/ele.14276

[B14] Ramírez MJ (2014) The morphology and phylogeny of dionychan spiders (Araneae: Araneomorphae). Bulletin of the American Museum of Natural History 390: 1–374. 10.1206/821.1

[B15] Ramos D Hartke TR Buchori D Dupérré N Hidayat P Lia M Harms D Scheu S Drescher J (2022) Rainforest conversion to rubber and oil palm reduces abundance, biomass, and diversity of canopy spiders. PeerJ 10: e13898. 10.7717/peerj.13898 PMC9390325 35990898

[B16] Simon E (1887) Espèces et genres nouveaux de la famille des Sparassidae. Bulletin de la Société Zoologique de France 12(2–4): 466–474.

[B17] Simon E (1897) Histoire naturelle des araignées. Deuxième édition, tome second. Roret, Paris, 1–192. 10.5962/bhl.title.51973

[B18] Tang G Li SQ (2010) Crab spiders from Hainan Island, China (Araneae, Thomisidae). Zootaxa 2369(1): 1–68. 10.11646/zootaxa.2369.1.1

[B19] Thorell T (1890) Diagnoses aranearum aliquot novarum in Indo-Malesia inventarum. Annali del Museo Civico di Storia Naturale di Genova 30: 132–172.

[B20] Thorell T (1891) Spindlar från Nikobarerna och andra delar af södra Asien. Kongliga Svenska Vetenskaps-Akademiens Handlingar 24(2): 1–149.

[B21] Wheeler WC Coddington JA Crowley LM Dimitrov D Goloboff PA Griswold CE Hormiga G Prendini L Ramírez MJ Sierwald P Almeida-Silva L Alvarez-Padilla F Arnedo MA Benavides LR Benjamin SP Bond JE Grismado CJ Hasan E Hedin M Izquierdo MA Labarque FM Ledford J Lopardo L Maddison WP Miller JA Piacentini LN Platnick NI Polotow D Silva-Dávila D Scharff N Szúts T Ubick D Vink CJ Wood HM Zhang J (2017) The spider tree of life: Phylogeny of Araneae based on target-gene analyses from an extensive taxon sampling. Cladistics 33(6): 574–616. 10.1111/cla.12182 34724759

[B22] Zhong Y Zheng MY Liu KK (2022) First description of the male of *Ibanagan* Liu & Li, 2022 from China. Acta Arachnologica Sinica 31(2): 141–146. 10.3969/j.issn.1005-9628.2022.02.014

